# No evidence for sex chromosomes in natural populations of the cichlid fish *Astatotilapia burtoni*

**DOI:** 10.1093/g3journal/jkad011

**Published:** 2023-01-17

**Authors:** Nicolás Lichilín, Walter Salzburger, Astrid Böhne

**Affiliations:** Zoological Institute, Department of Environmental Sciences, University of Basel, Vesalgasse 1, 4051 Basel, Switzerland; Department of Neuroscience and Developmental Biology, University of Vienna, Djerassiplatz 1, 1030 Vienna, Austria; Zoological Institute, Department of Environmental Sciences, University of Basel, Vesalgasse 1, 4051 Basel, Switzerland; Zoological Institute, Department of Environmental Sciences, University of Basel, Vesalgasse 1, 4051 Basel, Switzerland; Leibniz Institute for the Analysis of Biodiversity Change, Museum Koenig Bonn, Adenauerallee 127, 53113 Bonn, Germany

**Keywords:** sex determination, genome, GWAS, genome sequencing, cichlid fish, sex chromosomes

## Abstract

Sex determination (SD) is not conserved among teleost fishes and can even differ between populations of the same species. Across the outstandingly species-rich fish family Cichlidae, more and more SD systems are being discovered. Still, the picture of SD evolution in this group is far from being complete. Lake Tanganyika and its affluent rivers are home to *Astatotilapia burtoni*, which belongs to the extremely successful East African cichlid lineage Haplochromini. Previously, in different families of an *A. burtoni* laboratory strain, an XYW system and an XY system have been described. The latter was also found in a second laboratory strain. In a laboratory-reared family descending from a population of the species’ southern distribution, a second XY system was discovered. Yet, an analysis of sex chromosomes for the whole species distribution is missing. Here, we examined the genomes of 11 natural populations of *A. burtoni*, encompassing a wide range of its distribution, for sex-linked regions. We did not detect signs of differentiated sex chromosomes and also not the previously described sex chromosomal systems present in laboratory lines, suggesting different SD systems in the same species under natural and (long-term) artificial conditions. We suggest that SD in *A. burtoni* is more labile than previously assumed and consists of a combination of non-genetic, polygenic, or poorly differentiated sex chromosomes.

## Introduction

Sex determination (SD) is the process of a sexually reproducing organism defining its sex (Capel 2017). Albeit serving the unifying goal of establishing sex, SD systems and their molecular mechanisms are not conserved across the tree of life (Cutting *et al.* 2013; Bachtrog *et al.* 2014) and can even differ among populations of the same species (Anderson *et al.* 2012; Yamamoto *et al.* 2014; Li *et al.* 2018; Pennell *et al.* 2018; Taslima *et al.* 2020). For example, in the European common frog (*Rana temporaria*), a gradient of sex chromosome differentiation exists along a geographical range (Rodrigues *et al.* 2014); sex chromosomes exhibit higher genetic differentiation in the northern-boreal population of the common frog compared to the southernmost population, while populations in between show intermediate levels of sex chromosome differentiation at the sequence level. In the latter, Rodrigues *et al.* (2014) noted some mismatches between genotypic and phenotypic sex, suggesting that other mechanisms or factors could be driving SD or overriding the otherwise strong genetic sex determiner. Likewise, the Japanese wrinkled frog (*Rana rugosa*) has four types of SD systems depending on its region of origin (Miura 2008). In the North of Japan, *R. rugosa* has a female ZW heterogametic system with heteromorphic sex chromosomes, while the three southern/western forms feature a male XY heterogametic system, but only one of them has differentiated sex chromosomes. Finally, in a stickleback (*Gasterosteus aculeatus*) population from the Japan Sea, a neo-Y sex chromosome was identified that seems to be coupled to reproductive isolation (Kitano *et al.* 2009).

Interestingly, previous studies revealed that laboratory model species can show discrepancies in their sex chromosomal systems compared to those present in their wild counterparts: Laboratory strains of zebrafish (*Danio rerio*) typically lack cytogenetically detectable heteromorphic sex chromosomes and have been assumed to use a polygenic SD system (Amores and Postlethwait 1998; Phillips *et al.* 2006). However, a study performed on natural zebrafish populations identified a heteromorphic ZW sex chromosomal system (Sharma *et al.* 1998). Within some laboratory strains, although lacking signs of sex chromosome differentiation, crosses also suggested dominant female sex determiners (Tong *et al.* 2010). Furthermore, SD in zebrafish is sensitive to harsh environmental conditions and different studies revealed different sex-associated loci (Anderson *et al.* 2012; Liew *et al.* 2012; Howe *et al.* 2013; Liew and Orbán 2014), suggesting SD to be polygenic (Bradley *et al.* 2011; Liew *et al.* 2012). Finally, Wilson *et al.* (2014) showed that the ZW system found in natural zebrafish populations got “accidentally” lost, or selected against, during the establishment of laboratory lines when selecting against mildly deleterious mutations.

In Siamese fighting fish (*Betta splendens*), laboratory strains show higher penetrance for an XY system than their wild counterparts, probably due to selection during domestication in favor of more predictable sex ratios. Other sex determination systems might be at play in the wild (Kwon *et al.* 2022).

Observations mainly of laboratory-reared fish have revealed polygenic SD in Lake Malawi cichlids with different co-existing sex chromosomes and hence several male and female genotypes with epistasis between alleles at the different loci determining sex (e.g. Moore *et al.* 2022).

Here, we set out to investigate genomic signatures of sex linkage in natural populations of the cichlid fish *Astatotilapia burtoni* (Günther 1893). *A. burtoni* has served as model species in numerous research fields, including neurobiology (Hofmann 2003; Maruska and Fernald 2018), developmental biology (Robison *et al.* 2001; Juntti *et al.* 2016), behavior (Hofmann 2003; Theis *et al.* 2017), genetics and genomics (Lang *et al.* 2006; Egger *et al.* 2017; El Taher *et al.* 2019), and speciation (Weber *et al.* 2021). *A. burtoni* is phylogenetically placed within the “modern haplochromines” (Salzburger *et al.* 2005; Meyer *et al.* 2015). The modern haplochromines form the most species-rich cichlid tribe comprising the Lake Tanganyika (LT) endemic Tropheini and the entire species flocks of the Lake Malawi and Victoria radiations, among others (Verheyen 2003; Salzburger *et al.* 2005, 2014). While *A. burtoni* is present in LT, it is not part of the endemic LT radiation (Salzburger 2018; Ronco *et al.* 2020, 2021). *A. burtoni* occurs in LT, tributary rivers and swamps (De Vos *et al.* 2001; Kullander and Roberts 2011), with a high degree of population structure between the northern and southern part of the lake detected by nuclear markers, as well as a deep divergence between southwestern populations and populations from the north and southeastern part of the lake (Pauquet *et al.* 2018; Weber *et al.* 2021).

Sex-determining regions of *A. burtoni* have been identified on three different chromosomes (Table 1). Following the cichlid community convention, the naming of *A. burtoni* chromosomes refers to linkage groups of the genome assembly of another cichlid species, the Nile tilapia (*Oreochromis niloticus*). Cichlids show high degrees of large-scale chromosomal synteny (Mazzuchelli *et al.* 2012). The Nile tilapia is often used as a reference due to the quality of its reference genome (Conte *et al.* 2017), its karyotype of 2*n* = 44 [the modal karyotype of African cichlids, (Majtánová *et al.* 2019)], as well as the fact that it is an outgroup to all cichlid species of the great lake radiations (Ronco *et al.* 2021, Extended Data Fig. 1, divergence of Oreochromini ∼16 Mya).

**Fig. 1.**
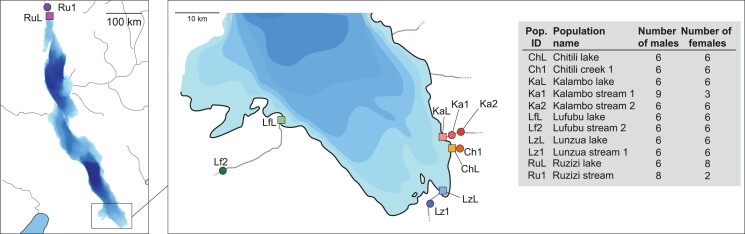
Map of Lake Tanganyika showing the 11 sampling locations of populations investigated. Squares represent lake, and circles represent stream populations. Bathymetric colors indicate lake depth (increasing color darkness represents increasing depth). Modified from Theis *et al.* (2017) with permission.

**Table 1.** *Astatotilapia burtoni* sex determination systems previously described in laboratory-reared specimens.

Reference	SD system type	Corresponding LG in *O. niloticus* genome	Genomic location on *O. niloticus* LG	SD candidate genes
Roberts *et al.* 2016	XY	LG05-14 (fused in *A. burtoni*)	LG05 0–25 Mb LG14 0–18 Mb	*wnt4* *wnt7a*
	XYW	LG13	3.2–7.7 Mb	*cyp17a1* *bmpr1a*
Böhne *et al.* 2016	XY	LG05-14 (fused in *A. burtoni*)	LG05 6.8–20.3 Mb LG14 0–15 Mb	*wnt4* *wnt7a*
	XY	LG18	3.9–19 Mb	—

In two *A. burtoni* laboratory strains, an XY system is located on LGs 05 and 14, supporting a chromosomal fusion of these LGs compared to the Nile tilapia karyotype and in line with the *A. burtoni* karyotype (Böhne *et al.* 2016; Roberts *et al.* 2016). In families of one of the laboratory strains, an XYW system was detected on LG13 (Roberts *et al.* 2016). In a laboratory-reared family derived from wild-caught individuals of the south of LT, an XY system was found on LG18.

The reported XYW region on LG13 spans 4.6 Mb at the beginning of LG13 with *cytochrome P450 17alpha-hydroxylase* (*cyp17a1*) and *type I bone morphogenetic protein receptor* (*bmpr1a*) as prime candidate genes for SD; The XY region on LG05/14 has the highest density of and most significantly sex-associated SNPs located on LG05 between 6.8 and 20.3 Mb, including *wingless-type MMTV integration site family member 4* (*wnt4*) as the top candidate gene for SD. The XY system on LG18 encompasses ∼16 Mb (Böhne *et al.* 2016). Until now, the interaction and evolutionary history of these three sex-linked systems remain unstudied, in particular in wild *A. burtoni* populations.

Here, we investigated signatures of sex-linkage based on genomic data of specimens of 11 natural populations of *A. burtoni* (Fig. 1). We searched the genomes of 61 females and 71 males for molecular traces of the three sex chromosomal systems identified previously (Böhne *et al.* 2016; Roberts *et al.* 2016). To this end, we performed calculations of intersex Fst and a genome-wide association (GWAS) test with sex. Next, we generated *de novo* genome assemblies for each sex of the 11 populations and performed coverage analyses to trace down differences between sexes. We further applied a k-mer-based approach to detect sex-associated regions across the genome and used those to confirm and narrow down previous genome-wide results. Finally, we tested the prevalence of previously described male-specific markers in the natural populations.

## Materials and methods

### DNA samples

We analyzed whole-genome sequencing data from Weber *et al.* (2021), accessible under the NCBI BioProject accession number PRJNA485198, from 11 different *A. burtoni* populations, comprising a total of 132 individuals. Genome data were available for typically six females and six males per population; for exact numbers, geographic locations, names, and acronyms of the populations used in this study, see Fig. 1.

### Variant calling

DNA-sequencing data per individual were quality filtered and adapters removed with Trimmomatic V0.36 (Bolger *et al.* 2014) in PE mode with the settings adapterfile:2:30:12:8:true MINLEN:30. Reads were mapped against the Nile tilapia (*Oreochromis niloticus*) genome assembly version 2 (RefSeq accession number GCF_001858045.1_ASM185804v2), which was the only cichlid genome assembly on chromosomal level available to us at the time. Prior to mapping, unplaced scaffolds of this genome assembly were concatenated lexicographically into an “UNPLACED” super chromosome. This customized reference was indexed with BWA V0.7.13 and individual DNA reads were aligned against it with bwa-mem under default parameters (Li and Durbin 2009). Alignments were coordinate-sorted and indexed with SAMtools 1.3.1 (Li *et al.* 2009). Variants were called with GATK’s V3.7 (McKenna *et al.* 2010) HaplotypeCaller (per individual and per chromosome), GenotypeGVCFs (per chromosome) and CatVariants (to merge all obtained VCF files). The final variants were filtered with GATK's VariantFiltration with settings “QD < 2.0”, “FS > 200.0”, “ReadPosRankSum < −20.0”, “SOR > 10.0”, “DP < 200” and “DP > 4,000” for indels and “MQ < 40.0”, “FS > 60.0”, “QD < 2.0”, “DP < 200”, “DP > 4,000”, ‘SOR > 7.5”, “MQRankSum < −12.5”, and “ReadPosRankSum < −10.0” for SNPs.

### Genome-wide association test for sex

From the initially filtered vcf file with all populations combined, we removed sites with more than 20% missing data and sites that had more than two alleles, retaining only SNPs. The obtained vcf was phased and genotypes were imputed with beagle V5 (Browning and Browning 2016) and transformed into bed format with PLINK V1.9 (Purcell *et al.* 2007; Chang *et al.* 2015). We then ran an association test for sex using the univariate linear mixed model LMM integrated in GEMMA V0.97 (gemma -notsnp -lmm 4) accounting for population stratification with a relatedness matrix calculated within GEMMA (-k option) (Zhou and Stephens 2012). Genotypes of potential sex-linked sites were visualized with the R package Pheatmap V1.0.12 in R V3.6.0 (R Core Team 2018) using vcfR V1.8.0 (Knaus and Grünwald 2017).

### Population genetic statistics

SNPs per population were subsetted from the filtered vcf file described above. We then calculated intersex Fst and male and female nucleotide diversity in windows of 10 kb with VCFtools V0.1.14 (Danecek *et al.* 2011).

### Per population *de novo* genome assemblies

In order to analyze per-population coverage differences between the sexes, we generated *de novo* draft genome assemblies for each sex and each population. To guarantee sufficient coverage, sequencing reads of two randomly selected individuals per sex for the 11 *A. burtoni* populations were combined as input for these 22 *de novo* draft genome assemblies. *A*ssemblies were generated as previously described (Malmstrøm *et al.* 2017; Böhne *et al.* 2019) using Celera Assembler V8.3 (Myers *et al.* 2000) and indexed with BWA V0.7.13 (Li and Durbin 2009). We aligned the draft assemblies against the Nile tilapia reference genome (refseq accession number GCF_001858045.2_O_niloticus_UMD_NMBU) using LAST V861 and lastal (Kiełbasa *et al.* 2011) to infer chromosomal locations of draft genome scaffolds.

### Sequence coverage analyses per population

Following the strategy described by Böhne *et al.* (2019), we assessed differences in sequence coverage between males and females of a population to identify sex-linked genomic regions under the expectation that Y- and W-specific regions are not present in females and males, respectively, and that coverage on X and Z in sex-differentiated regions are reduced in males and females, respectively. To this aim, reads of each individual were trimmed and quality filtered prior to aligning them to the two *de novo* assemblies of the corresponding population, as described above (see variant calling section). Sequencing depth from all mapped reads per individual was calculated with SAMtools for all positions (i.e. samtools depth -aa). We next calculated the median of coverage per sex per population for each *de novo* assembly in R V3.5 (R Core Team 2018).

To visualize global patterns of coverage difference between males and females of a given population based on the method described in Böhne *et al.* (2019), we ordered scaffolds of each *de novo* assembly based on the coordinates retrieved from LAST alignments against the Nile tilapia reference genome. In addition, to extracting coverage from all alignments (which represents mixed coverage from both gametologs in the heterogametic sex if the reference genome only contains one sex chromosome and/or gametologs are poorly differentiated), we also retained only alignments with no mismatch (i.e. keep reads with flag “NM:i:0”) from reads mapped in proper pairs of the BAM files for each sex and population with SAMtools (i.e. samtools view –f 2). We estimated sequencing depth for those files in the same way described above, per sex and per population and as previously done in Böhne *et al.* (2019). Depth for males and females was visualized in 10 kb windows in R, as log2[(median values per sex per site)/(median of depth for the whole genome)]. To obtain genomic windows with significant differences in sequencing depth between sexes within linkage groups, we calculated the median male-female coverage ratio and median male-female coverage difference for each window, after depth normalization (i.e. median values per sex per site/median of depth for the whole genome). We identified windows with median-normalized coverage ratios outside the 99% quantile and a median-normalized coverage difference between the sexes >0.25 (i.e. the minimum value of coverage twice as high as the coverage in the opposite sex). Subsequently, we assessed statistical significance for each of the retained windows with a two-sided Wilcoxon signed-rank test for the median-normalized coverage values between sexes, with the corresponding 95% confidence interval. Finally, to test for differences in the number of significant windows with reduced coverage found for each assembly across chromosomes, a one-sided Fisher's exact test and Bonferroni correction for multiple testing were performed in R.

To identify sex-limited regions (i.e. Y- or W-chromosome sequences) in the *de novo* assembled scaffolds, we identified regions having (i) zero mapping coverage in the sex contrasting the one used in the assembly and (ii) mapping coverage of zero in no more than one individual of the same sex as used for the assembly.

We retained all regions with such a coverage pattern and a minimal length of 500 bp. Sex-specific median-normalized coverage of scaffolds containing one or more of these 500 bp regions were extracted, and coverage was visualized in 100 bp windows with the R package tidyverse (Wickham *et al.* 2019). Sequence similarities among scaffolds containing the so-identified sex-specific regions across all populations within each sex were assessed using the CAP3 sequence assembly program (Huang and Madan 1999) with default parameters, which collapses contiguous sequences. Next, we assigned sex-specific scaffolds to genomic regions of the Nile tilapia reference genome based on the LAST alignments described above. Finally, to identify protein-coding sequences in sex-specific regions, we extended the sequence of the sex-specific regions into their flanking regions to a total length of 5 kb (whenever contig length permitted) and blasted the obtained sequences to the NR database (version 2018-10-23) with blastx BLAST + V2.7.1 (Camacho *et al.* 2009). We retained the top 20 alignments with an e-value cutoff of <0.001.

### Heterogametic system detection with k-mers

We applied a second approach to identify sex-specific sequences and their location in the genome as described in Akagi *et al.* (2014) and Böhne *et al.* (2019) for 10 of the 11 populations. We excluded the Ruzizi River population due to the low number of female samples (Fig. 1). Starting from trimmed sequencing reads (see above), we generated k-mer catalogs per population of all possible k-mers starting with “AG” and a length of 37 bp present in at least five specimens per population using a Python script provided in Akagi *et al.* (2014). We divided k-mer catalogs into four categories: Y-k-mers = male-specific, Z-k-mers = male-biased, X-k-mers = female-biased, and W-k-mers = female-specific. To this end, we applied a linear regression to the k-mer counts of each population and retained outliers from the general distribution by calculating studentized residuals from a linear model (i.e. jack-knifed residuals). Outliers were defined as all k-mers with an absolute studentized residual value equal to or bigger than 3, as an observation with an absolute value of 3 is deemed to be an outlier (Belsley *et al.* 1980; Hettmansperger 1987; Atkinson 1994). Subsequently, sex-specific k-mers (i.e. Y- or W-k-mers) were defined as k-mers having zero counts in one sex but not in the opposite sex. Sex-biased k-mers were obtained based on the ratio of counts between males and females, expecting larger counts for the homogametic sex (e.g. X-k-mers = female count/male count > 4, depending on the population analyzed). In summary, we retained outlier k-mers from the linear regression and from there we took (i) sex-specific k-mers (either Y- or W-k-mers) and (ii) sex-biased k-mers with ratios bigger than four for all populations but not in Kalambo River 1, (i.e. ratio threshold set to 12 for Z-k-mers due to the lower number of female samples for this population, see Fig. 1). Next, we tested for an increased amount of sex-specific k-mers per population with a Wilcoxon test, aiming to detect the heterogametic sex of the population. Additionally, we identified k-mers shared among populations in each category with UpSetR (Conway *et al.* 2017) in R.

For the Kalambo River (Ka2) and Chitili River (Ch1) populations, we extracted sequencing reads and their mates containing Y-k-mers of each population. Next, we assembled the extracted reads with MEGAHIT (Li *et al.* 2015) with *–k-max* 12. We also placed the resulting contigs onto the Nile tilapia reference genome with BWA and compared the contig data sets using blastX to the NR database in Blast2GO (Gotz *et al.* 2008) to retrieve functional annotations.

### Population phylogenies using k-mers

To investigate if there are k-mers that would group individuals within or across populations by sex rather than reflecting the population structure, we computed phylogenies per population, for all samples, and for all samples within each chromosome using specimen k-mer counts generated with the alignment and assembly-free (AAF) software (Fan *et al.* 2015). We performed this calculation with two k-mer sizes, the default k-mer size of 25 bp and more specific 31 bp k-mers.

### Comparing Y-specific sequences from a laboratory population

Using previously obtained and located (LG05) male-specific sequences from a restriction site associated DNA-sequencing experiment (Böhne *et al.* 2016), we tested for sequence similarities of these Y-specific RAD tags to the *de novo* assemblies with BlastN (Camacho *et al.* 2009).

### Further sex chromosome identification approaches

We implemented SEX-DETector in a population through a Bayesian approach (SDpop) (Käfer *et al.* 2021) and Sex Assignment Through Coverage (SATC) (Nursyifa *et al.* 2022) per population and across all samples to detect sex chromosomes. These methods have been used to detect young and mildly degenerated sex chromosomes across different taxa.

## Results

### Intersex population genetics, sex association and previously identified sex-linked sites in A. burtoni natural populations

Similar to other studies (Wilson *et al.* 2014; Conte *et al.* 2017; Bergero *et al.* 2018; Pan *et al.* 2019), we searched for an accumulation of sex-specific alleles with the expectation that a sex chromosome would show increased sequence differences between males and females. Based on intersex Fst and comparisons of male-female nucleotide diversity within each population, we did not detect any sex-differentiated chromosome [i.e. expected increase of intersex Fst to 0.5 (Bhatia *et al.* 2013)] on the population level (Supplementary Fig. 1 File 1).

**Fig. 2.**
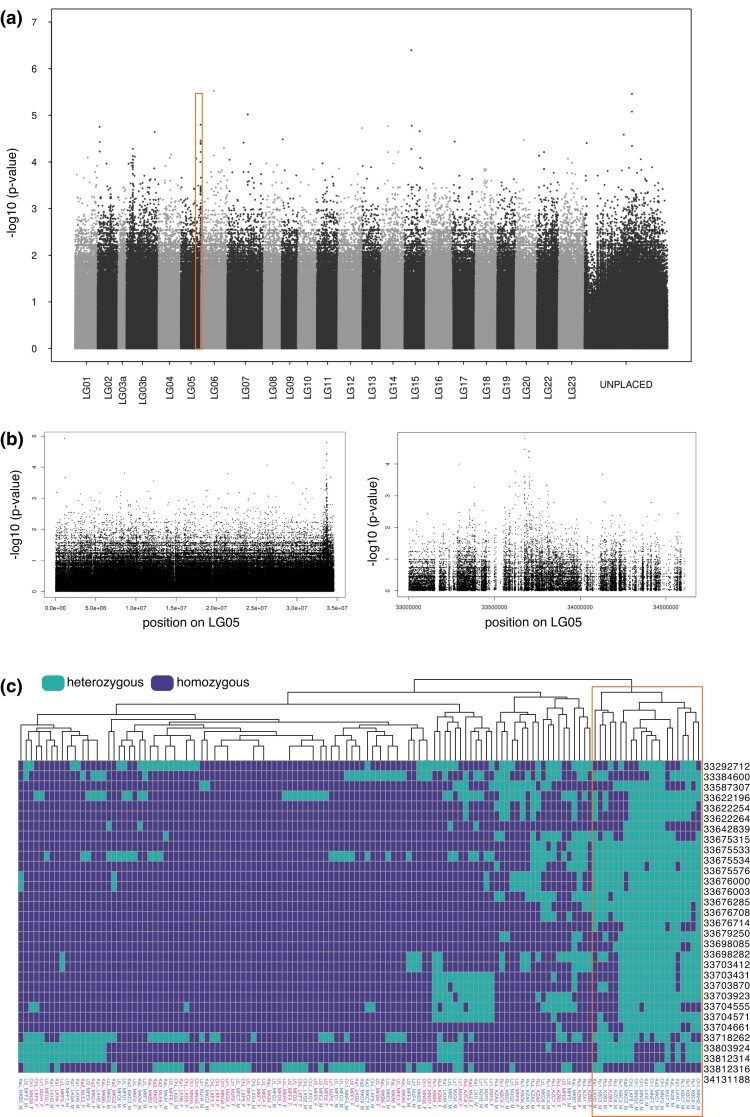
a) Manhattan plots of a GWAS analysis for an association with sex using the reference genome of the Nile tilapia (*Oreochromis niloticus*). Interchanging black and gray colors delimit chromosomes. Unplaced scaffolds were concatenated into an “UNPLACED” chromosome for visualization. The overlayed box indicates a region on LG05 with concentrated and increased association to sex. b) Magnifications of the region on chromosome 5 depicted with a box in panel a. c) The heatmap shows individual genotypes for SNPs with an elevated association to sex in a narrow peak region detected on LG05. A group of male individuals is largely heterozygous in this region (green squares) and groups by sex and not population (males from Ch1, Ru1, RuL, KaL, Ka1, and Ka2 populations; highlighted with a box). Chromosomal coordinates are shown on the right side; the sample clustering tree is shown on top; samples are indicated below the plot: _M for males and _F for females. Green: heterozygous genotypes, dark blue: homozygous genotypes.

A GWAS analysis based on a dataset combining SNPs of all specimens of all populations did not identify a region of elevated, significant sex association. Concerning the previously identified sex-linked LGs in laboratory strains (Böhne *et al.* 2016; Roberts et al. 2016), visual inspection revealed only a narrow and not drastically elevated peak of SNPs with association to sex at the end of LG05 (Fig. 2a). Since this chromosome has previously been implicated in sex determination not only in *A. burtoni* but also other haplochromine cichlids (Böhne *et al.* 2016; Böhne *et al.* 2019; El Taher et al. 2021), we inspected the genotypes of the SNPs in this region. This revealed an excess of SNPs specific almost exclusively to a group of male samples from different populations (one female was included in this group as well as 20 males), matching an XY-patterning however only in a subset of samples and not reaching usual significant levels applied in GWAS (Fig. 2b and c, males from Ch1, Ru1, RuL, KaL, Ka1, and Ka2 population).

Furthermore, while the chromosomal association would be consistent with previous results from *A. burtoni*, the exact position of these potentially male-specific SNPs differed from previous studies with a sex-determining region located at the opposite end of LG05 (Böhne *et al.* 2016).

We next inspected male and female genotypes of all individuals of genomic regions previously shown to be associated with sex (Böhne *et al.* 2016; Roberts *et al.* 2016). We did not observe any obvious clustering of sample genotypes by sex, which would have been indicative of an excess of heterozygous sites in one sex (e.g. males in the XY regions or females in the ZW region). Instead, we recovered the phylogenetic split between the southern and northern populations and mostly among the southern populations (Supplementary File 2).

We next investigated the presence of previously identified male-limited Y-specific sequences from a RAD-sequencing experiment of an *A. burtoni* laboratory strain in the population genomic data. Y-specific RAD markers identified from a laboratory strain were present in almost all male and female genome assemblies (Supplementary Table 1). Only one of the markers was not present in the female assemblies of two southern populations, ChL and Ka2 (Supplementary Table 1). Using a projection of our *de novo* assembled scaffolds to the Nile tilapia genome, we confirmed that the RAD tag sequences were located on scaffolds matching to LG05. For four of the assemblies, however, the scaffolds presented multiple mapping matches of similar quality also to other chromosomes (Lf2 female assembly to LG06, in ChL female assembly to LG03, in Ka1 male assembly to LG16 and in LfL female assembly to LG18). This suggests possible sequence duplications in the genome.

### Identification of sex differences in coverage across the genome

We next targeted differences in coverage between sexes based on newly generated sex-specific *de novo* assemblies of each population. The quality analysis of the generated *de novo* assemblies revealed consistent and thus comparable quality across assemblies (Supplementary Table 2). We additionally tested for differences in the back-mapping quality within populations for the *de novo* assemblies (Supplementary Fig. 2 File 1 and Table 3), revealing a high average back-mapping rate (96.4–97.6%, Supplementary Table 3). There were no significant global differences in mapping and coverage between males and females within the same population, supporting no sex-specific skew in sequencing nor mapping (Supplementary Fig. 2 File 1). We did not detect sex-specific, chromosome-wide patterns of low sequence coverage in any of the populations (Supplementary File 2), which once more supports the result that there is no (strongly) differentiated sex chromosome in these populations.

**Fig. 3.**
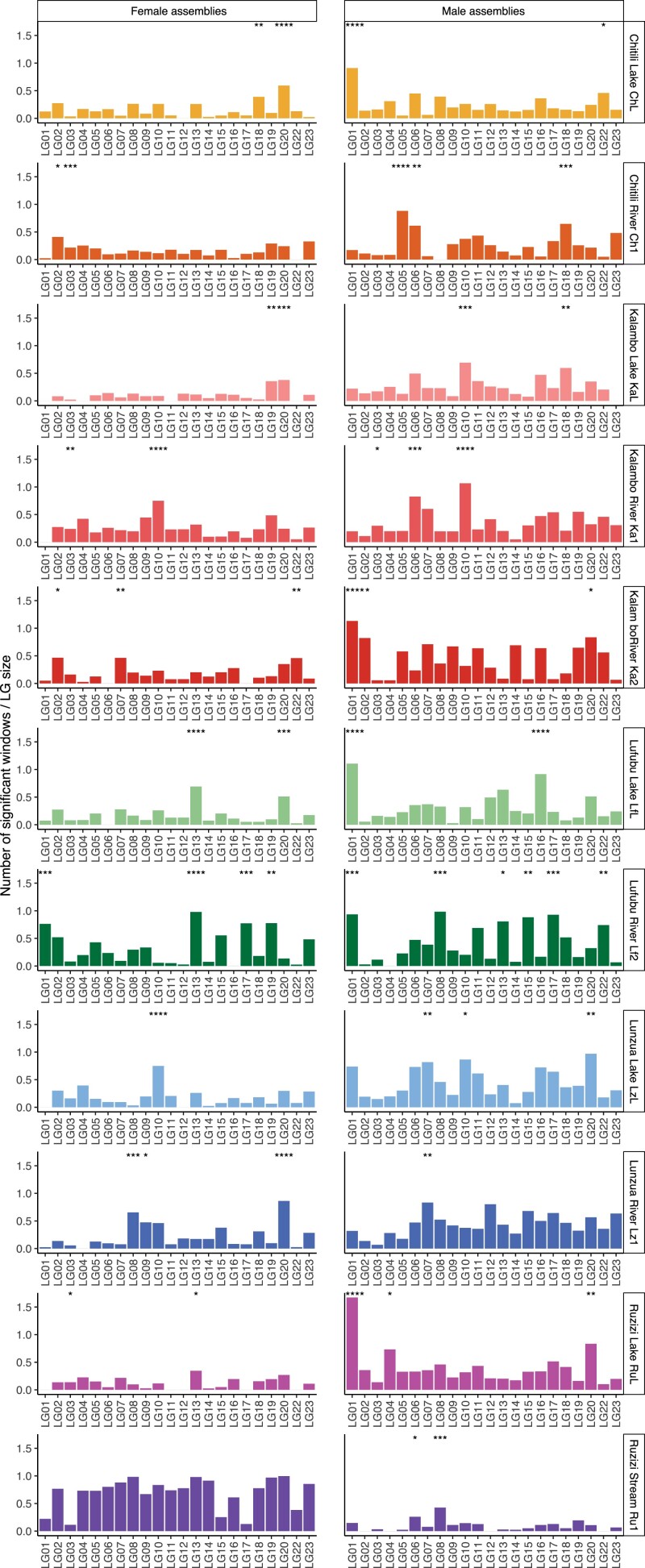
Reduced coverage based on mapping to sex-specific genome assemblies per chromosome (LGs). Bar plots show the number of 10 kb outlier windows of reduced coverage in males when mapped to female assemblies (left side) and in females when mapped to male assemblies (right side) by population and normalized by chromosomal length. Populations are color-coded as shown in Fig. 1. Significance levels after Bonferroni correction of a one-sided Fisher's exact test comparing the number of significant windows detected per each chromosome within a population and sex are indicated with *P ≤ 0.05, **P ≤ 0.01, ***P ≤ 0.001, ****P ≤ 0.0001.

We next identified 10 kb windows on each chromosome in each assembly with reduced sequence coverage in the opposite sex of the reference assembly (Supplementary Table 4) and tested if the number of such windows differed among chromosomes in each assembly (Fig. 3 and Supplementary Table 5). Assuming an XY system with sequence differentiation between X and Y, we would expect a reduced coverage in males for X-linked regions using the female assemblies, and *vice versa* a reduced coverage in females for Z-linked regions when using the male assemblies (Fig. 3). In poorly differentiated sex chromosome systems, we would, however, expect some read mapping to the Y chromosome from X-chromosome-originated reads as well as to the W-chromosome from Z-linked reads. Hence, we might not be able to unambiguously determine the type of heterogamety with this approach.

Concerning the previously described sex chromosomal system, we found little to no support for sex chromosome differentiation with this approach (Fig. 3 and Supplementary Table 5). For the two known XY systems, solely LG18 showed significantly reduced male coverage in the female assembly of ChL (which would support an XY system) but even more so on LG20. LG13 showed decreased female coverage only in Lf2 for male assemblies (supportive of a ZW system); however, several other LGs did as well. Interestingly, in the Ch1 male assembly, we detected significantly reduced female coverage on both LG05 and LG18. On LG05, most of the windows were located at the beginning of the chromosome (Supplementary File 3). As indicated above, while this might support loci with a ZW pattern, it could also be caused by Y-linked regions that still show some similarity to their X-linked alleles and, hence, have a poorer mapping in females. Out of all chromosomes that had previously not been described as sex-linked in *A. burtoni*, particularly, LG01 showed significantly reduced female mapping-coverage in five populations (ChL, Ka2, LfL, Lf2, RuL) and LG20 showed reduced male mapping-coverage in female assemblies in four populations (ChL, KaL, LfL, Lz1).

Based on the female assemblies, the two Lufubu populations (LfL and Lf2) showed a high number of windows of reduced male coverage that could support X-chromosome-like windows on LG13. The same pattern was true for the RuL population, albeit with a lower significance level (Fig. 3 and Supplementary Table 5). Overall, cichlid sex chromosomes are not strongly degenerated and hence the power to detect sex chromosomal sequences over coverage differences is limited, potential signals might be increased by increasing sample sizes but still be obscured if several SD systems coexist in *A. burtoni*.

### Identification of sex-limited sequences

We next identified genomic regions from the *de novo* assemblies with regions of at least 500 bp length with zero read mapping in the sex not used for the assembly (Table 2 and Supplementary File 4). The number of these presumably sex-specific regions within the populations was rather low (0 to 56, average = 6.5) (Table 2). Some sex-specific regions were located within the same scaffold (Table 2 and Supplementary Fig. 3a & b File 1). The population that showed the largest number of female- and male-specific regions was Ru1, while Ka2 also showed an increase in male-specific regions; this suggests that Ru1 and Ka2 could have male sex-determining regions that are differentiated to some extent. However, it is important to keep in mind that sampling is sex-biased in the Ruzizi River population. We did not find a shared sex-specific region across populations within assemblies, supporting the hypothesis that there is no general conservation of sex chromosomes in *A. burtoni*. The number of sex-specific regions could lend some support for a ZW system in ChL and Lz1.

**Table 2.** *De novo* assembled male and female scaffolds containing sex-specific regions (SSRs).

Assembly sex	Population	Nr. of scaffolds in assembly	Nr. of SSR larger than 500 bp	Nr. of scaffolds with SSRs larger than 500 bp	95% confidence intervals for the scaffolds with SSRs larger than 500 bp
**Female**	ChL	46,282	6	5	1.62–11.67
	Ch1	47,268	2	2	0.24–7.22
	KaL	49,070	1	1	0.03–5.57
	Ka1	46,494	5	4	1.09–10.24
	Ka2	48,912	2	1	0.03–5.57
	LfL	46,346	3	3	0.62–8.77
	Lf2	48,402	1	1	0.03–5.57
	LzL	47,122	1	1	0.03–5.57
	Lz1	51,131	10	6	2.2–13.06
	RuL	47,758	0	0	0–3.69
	Ru1	46,890	18	15	8.4–24.72
**Male**	ChL	47,835	0	0	0–3.69
	Ch1	47,714	2	2	0.24–7.22
	KaL	47,992	0	0	0–3.69
	Ka1	46,952	2	2	0.24–7.22
	Ka2	48,541	24	14	7.66–23.48
	LfL	46,613	1	1	0.03–5.57
	Lf2	48,243	1	1	0.03–5.57
	LzL	47,983	2	2	0.24–7.22
	Lz1	49,422	4	1	0.03–5.57
	RuL	47,334	2	2	0.24–7.22
	Ru1	45,710	56	39	27.74–53.3

Next, we inferred the coding potential for the scaffolds carrying the sex-limited regions and projected the scaffolds onto the Nile tilapia reference genome (Supplementary Fig. 3 File 1 and Supplementary Table 6). LG03 was the chromosome with the largest number of scaffolds with male-and female-limited regions (Supplementary Fig. 3 File 1 and Supplementary Table 6). This might be due to the fact that LG03 is the largest chromosome in the Nile tilapia genome. Note, however, that LG03 has also been reported as the sex chromosome in Oreochromini (Gammerdinger and Kocher 2018) and is rich in repetitive element content (Conte *et al.* 2017; Tao *et al.* 2020). Concerning the previously identified *A. burtoni* sex chromosomes, solely a female-specific region on a scaffold of Lf2 was located on LG13. However, we could not detect a coding gene in this region. Ka2 had one male-specific scaffold located on LG14 (Supplementary Fig. 3b File 1, Supplementary Table 6), yet, this scaffold also had secondary matches to different chromosomes (as LG05) and unplaced genomic regions (Supplementary Table 6). This scaffold had a protein sequence description associated with signaling receptor binding and regulation of immune response functions. Overall, no clear dominance on a single LG of the few sex-linked scaffolds was identified (Supplementary Fig. 3b File 1).

As a summary of the coding potential function detected, nine of the inferred protein-coding sequences showed similarities to mobile elements (6 scaffolds in females and 14 scaffolds in male assemblies), which are indeed a characteristic feature of sex chromosomes (Supplementary Fig. 3c File 1, Supplementary Table 6). Besides, several sex-limited sequences had a common protein-coding description between males and females. Most of the sequences were related to transposable elements as RNA-directed DNA polymerase, others were associated to small structural molecules such as zinc finger, and others were related to the immune system such as NACHT, LRR, and PYD domains or GTPase IMAP family members, among other associations (Supplementary Fig. 3c File 1, Supplementary Table 6). Finally, we did not find similarities to proteins that have previously been associated with SD or sex differentiation.

### K-mer approaches to detect heterogametic sex and sex-specific sequences

**Fig. 4.**
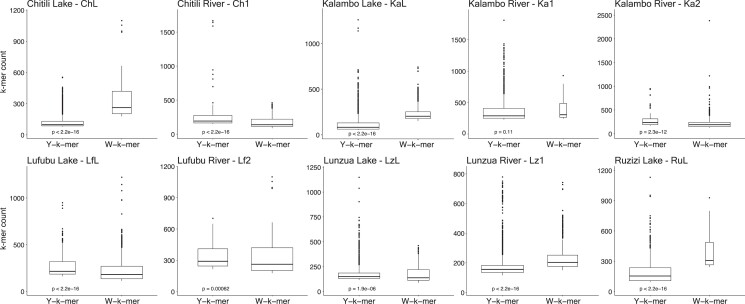
Boxplots of sex-specific k-mer coverage (Y- and W-k-mers) in the *A. burtoni* natural populations. Box width indicates the number of k-mers identified and the Y-axis their coverage. Wilcoxon test *P*-values are shown below for each population.

**Fig. 5.**
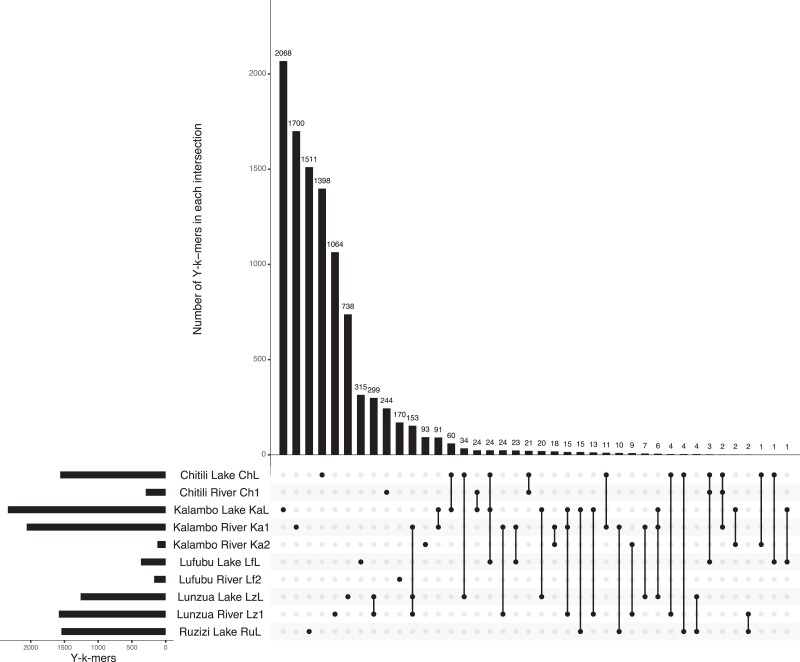
Comparison of Y-mers across populations. Number of male-specific k-mers per population is depicted by left-side bars. The compared populations are shown with dots (lower right) and the number of Y-k-mers found in each comparison is shown with bars. Note that there is a large proportion of k-mers specific to each population.

Since we did not detect major global sex differences along LGs, we next implemented a reference-genome-free approach by establishing k-mer catalogs for females and males of each population (Akagi *et al.* 2014; Böhne *et al.* 2019). Note that we excluded the Ru1 population due to the low number of female specimens available. We divided the k-mer counts of each population into categories, in either sex-specific (i.e. k-mers with zero counts in one sex, Y-mers or W-mers), or sex-biased (X- or Z-k-mers, see Supplementary Fig. 4 File 1 for a representation). We tested for a difference in Y-mer and W-mer coverage within each population as an indicator of a prevailing heterogametic system. Populations Ch1, Ka2, LfL and Lf2, and LzL had significantly higher coverage of Y-k-mers than W-k-mers (Fig. 4), supportive of male XY heterogametic systems. Conversely, ChL, KaL, Lz1, and RuL showed significantly higher coverage of W-k-mers than Y-k-mers, while having fewer k-mers overall (Fig. 4), suggesting a female ZW heterogametic system. Finally, we tested whether there were sex-specific and sex-biased k-mers shared across populations. Within each k-mer category, we found some degree of overlap between the k-mers of closely related populations; however, most k-mers were specific to a given population (Fig. 5 and Supplementary Fig. 5 File 1). Likewise, intersecting sex-specific and sex-biased k-mers showed that some k-mers belonged to opposite categories depending on the population investigated (e.g. 231 W-k-mers in KaL and Ka2 shared with Y-k-mers in LzL and Lz1, Supplementary Fig. 6 File 1). Finally, due to the fact that several analyses could support the presence of an XY male heterogametic system in Ka2, we further extracted reads containing Y-k-mers, assembled them and positioned the obtained contigs onto the Nile tilapia reference genome. We assembled 16 contigs, of which only two contigs had a location in a previously described sex-linked region, located in intergenic regions of LG13.

### Phylogenetic reconstructions using k-mer differences

Next, based on the presence and coverage of k-mers across samples, we tested whether specimens within and between populations would group by sex rather than following the species tree, which would be indicative of shared/ancestral sex chromosome sequences (Böhne *et al.* 2019). As presumed, the k-mer-based phylogeny largely recovered the species/population topology (Supplementary Fig. 7a, b File 1), previously described by Egger *et al.* (2017); Pauquet *et al.* (2018); Weber *et al.* (2021), since most k-mers are not expected to be located on sex chromosomes. Exploring the tree topologies in more detail within each population revealed that the topologies are consistent with using all specimens together. In none of the cases, specimens grouped unambiguously by sex (Supplementary Fig. 8 File 1). These results would confirm a small number of sex-specific k-mers across the whole genome in each *A. burtoni* population.

We further investigated whether, for any chromosome, including the previously described sex chromosomes, specimens would group by sex. To this aim, we divided the k-mer catalog into the corresponding chromosome in Nile tilapia (Supplementary Fig. 9 File 1). As in the genome-wide analysis, we did not find any clustering by sex based on chromosome-specific k-mers but rather a similar general phylogenetic grouping as using genome-wide k-mers.

### Further sex chromosome identification approaches

Additionally, we explored two recently published methods for SD detection that have successfully identified (young and mildly degenerated) sex chromosomes in other species: SATC (Nursyifa *et al.* 2022) and SDpop (Käfer *et al.* 2021). However, none of these novel methods detected any sex chromosome signal across all samples, nor within populations.

## Discussion

In this study, using a suite of genomic tools, we investigated potentially sex-linked genomic regions of wild *A. burtoni* populations of LT and its surroundings and compared novel genomic sequences to previous results obtained from laboratory and wild strains. Our analyses with respect to potential sex-linked regions provide little support to the findings of previous studies on the sex-linked regions of *A. burtoni* laboratory strains and a wild population (Böhne *et al.* 2016; Roberts *et al.* 2016). Overall, we did not detect a shared sex chromosome system across all populations, nor strong population-specific sex chromosome signals.

On the per population level, our diverse set of approaches might support a male heterogametic XY system in the Ch1, Ka1, and Ka2 populations; however, we could not unambiguously locate this signal to chromosome level. With the samples at hand, we suspect the presence of female heterogametic systems in ChL and Lz1. However, a more extensive sampling per population or focusing on single families derived from these populations is needed to further disentangle if and which genomic regions are truly sex-linked in *A. burtoni*.

Across populations, we detected some signal of a potential XY system on LG05 (in populations ChL, Ch1, KaL, Ka1, Ka2, RuL, Ru1) that does, however, not overlap with the previously identified sex-differentiated region of *A. burtoni* laboratory strains on this chromosome. LG05 is among the chromosomes that emerged multiple times independently as a sex chromosome in different African cichlid lineages (El Taher *et al.* 2021). In the future, the inclusion of more samples per population, family-based data, and a detailed analysis of the sex-linked regions on this chromosome might shed light on the underlying genes driving the convergent evolution of the same chromosome as the sex chromosome in several cichlid species.

Applying similar methods as we did here to other (cichlid) species (Akagi *et al.* 2014; Böhne *et al.* 2019; El Taher *et al.* 2021), the detection and characterization of sex-linked regions are possible. Consequently, potential sex chromosomal system(s) in *A. burtoni* natural populations seem to show little, if any gametolog differentiation, rendering the applied approaches powerless even with a total of 132 individuals. This can either be attributed to the fact that sex chromosomes, if at all present, are of young age and/or show ongoing recombination (Charlesworth 2017). “Old” sex chromosomes might appear undifferentiated under continuous recombination as postulated under the “fountain-of-youth” model (Perrin 2009), which is based on the recombination of sex chromosomes in sex reversals. However, an assumption of this hypothesis is strong heterochiasmy between the sexes, for which we lack evidence in cichlids. Still, we did not find evidence for suppressed recombination on any chromosome in one sex, and thus continued recombination among most of the *A. burtoni* genome in both sexes seems to be in place.

Another complementary explanation to our results is that SD in *A. burtoni* is a complex polygenic feature as previously proposed for an *A. burtoni* laboratory strain with different SD systems and alleles thereof in different families: XY on LG05/14 and XYW on LG13 (Roberts *et al.* 2016). Within some of the populations, we found support for both types of heterogamety. To understand if this is a sampling or sequencing artifact or indeed an indication of complex SD, an even more extended sampling of *A. burtoni* will be needed. Currently, since we could not unambiguously identify sex-linked loci, we cannot resolve the genetic architecture of SD in *A. burtoni*. Yet, if previously identified, on the population level co-existing two sex chromosomal systems (XYW on LG13 and XY on LG05/14) were indeed the sole possibilities, we should have picked up those regions when analyzing all populations together and probably also with the population-specific analyses given the 132 samples included here, which were sequenced to an average coverage per individual of 9.8× to 24.5× (Weber *et al.* 2021). Certainly, on the per population level, our power to detect a polygenic SD system is limited. While polygenic SD mechanism might occur in *A. burtoni* as it has also been reported for other cichlids (Ser *et al.* 2010; Liew *et al.* 2012; Moore *et al.* 2022), it is also possible that different *A. burtoni* laboratory strains display genetic mechanisms to determine sex not common in the wild, as supported by the previous finding of an XY system on LG18 in a wild population (Böhne *et al.* 2016) not found in laboratory strains. Our findings of multiple small regions that show sex association or are sex-limited to some degree may also suggest a polygenic SD system in *A. burtoni*, which may differ among strains and localities.

Our population data might support the hypothesis that SD in *A. burtoni* is indeed polygenic and probably involves more loci than previously thought. To resolve this scenario, further analyses of an even broader set of samples, including families would be needed. Analyses on the family or single population level, however, might fail to reveal the full complexity of SD of *A. burtoni*. Our data could also lend some support to the expectation that a polygenic system is unstable past a couple of generations (Rice 1986); polygenic SD is debated to represent a stable state (van Doorn and Kirkpatrick 2010) and could rather reflect a particular situation of sex chromosome turnover (Schartl *et al.* 2016).

It is also possible that SD in *A. burtoni* is leaky and dependent on geographic or ecological gradients, as we have shown for another haplochromine cichlid, *Pseudocrenilabrus philander* (Böhne *et al.* 2019). Similar scenarios have been described in the European frog (Rodrigues *et al.* 2014), the Japanese frog (Miura 2008), and to some extent in the Japanese stickleback (Kitano *et al.* 2009), which show an association between the SD system and the geographical location within a species. Nevertheless, we did not detect any sex-associated patterning in correlation to the *A. burtoni* habitat range.

As in many teleosts (and other species), the sex of *A. burtoni* can be reversed by interference with sex hormones (Heule *et al.* 2014). It is thus also possible that under natural conditions, SD in *A. burtoni* is less dependent on genetic factors but more so on the environment.

It is also possible that *A. burtoni* populations are in a transitioning state from one SD system to another. In general, sex chromosomes evolve rapidly in cichlids (Roberts *et al.* 2009; Ser *et al.* 2010) and indeed show frequent turnovers (El Taher *et al.* 2021).

Our results could further suggest that the easily identifiable and strongly sex-linked regions in the laboratory strains might result from an initial unintended sampling bias/founder effect or some inadvertent environmental modifications during laboratory handling and probably have been further selected for as a byproduct of selection under artificial breeding conditions (e.g. selection for early breeding, breeding of individuals at much smaller body size, early development of male-specific markings, among others).

Interestingly, data from fighting fish show a similar pattern as we identified, with a stronger prevalence of a simple sex chromosomal system in domesticated strains and less penetrance of this system in the wild (Kwon *et al.* 2022). Previous studies that characterized the SD system of zebrafish (*Danio rerio*) and the variation of SD mechanisms between natural and laboratory strains (Tong *et al.* 2010; Bradley *et al.* 2011; Wilson *et al.* 2014) revealed results in the opposite direction. Zebrafish wildtype populations have a ZZ/ZW system that lab strains have seemingly lost (Tong *et al.* 2010; Wilson *et al.* 2014).

The samples available to us cover the geographical distribution range of *A. burtoni* with representatives from 11 different populations across LT. While for two populations (i.e. Ka1 and Ru1), we had fewer female specimens than for the other populations that dataset was overall balanced; we further excluded biased populations in some analyses to minimize an effect of uneven sample sizes. Generally, the data include more representatives of the southern basin than the northern basin, a factor that could have an impact on the observed patterns and, therefore, might ask for a more in-depth analysis of more populations and specimens of the northern part of the LT basin, especially since the laboratory populations used in previous studies are derived from northern Ruzizi populations (Pauquet *et al.* 2018).

Our (laboratory) male-specific RAD tag marker analyses confirmed the genetic complexity in *A. burtoni* SD, as in some populations the location of the LG05 RAD-tags differed, suggesting different mechanisms of SD in those populations. We could not detect a distinctive, unique pattern of a simple sex-linkage in *A. burtoni* natural populations, while RAD data of laboratory strains identified clearly differentiated regions between gametologs. We suggest that *A. burtoni* SD in wild populations has not faced the same selective pressures as SD of specimens under laboratory rearing conditions; and while there could be a polygenic SD system in some laboratory strains and also in natural populations (though probably involving other or more loci), it is also possible that non-genetic factors affect SD or that sex chromosomes in *A. burtoni* are only very little differentiated. This opens the window to more in-depth studies of sex chromosomes in *A. burtoni* natural populations. For instance, assessing the sex ratio among several families might reveal the strength of the genetic component in *A. burtoni* SD (Liew *et al.* 2012); the more in-depth study of additional northern populations might show similarities in the SD system for laboratory stocks that were actually derived from these (Roberts *et al.* 2016; El Taher *et al.* 2019); genetic linkage mapping and association studies with larger numbers of specimens might identify particularly small sex differences as has been shown with a single fixed SNP difference between males and females of the pufferfish (Kamiya *et al.* 2012); and finally might reveal if there are instances of a mismatch between genotypic and phenotypic sex in *A. burtoni* natural populations indicative of sex reversal resembling the pattern observed in the European common frog (*Rana temporaria*) (Rodrigues *et al.* 2014).

Our shortcoming in detecting a sex-linked region in *A. burtoni* natural populations suggests that the SD system in *A. burtoni* is more unstable and prone to change and might involve more modifier loci than previously anticipated. Furthermore, we propose that the *A. burtoni* laboratory strains have undergone particular selection pressures under captive (in)breeding that might have given rise to the stronger differentiation of sex chromosomes. In general, sex chromosomal systems in teleost fish are labile (Kikuchi and Hamaguchi 2013; The Tree of Sex Consortium 2014), which might be perfectly exemplified in *A. burtoni*.

## Supplementary Material

jkad011_Supplementary_Data

## Data Availability

The raw data used in this study are derived from (Weber *et al.* 2021), accessible from the NCBI under the BioProject accession number PRJNA485198. All data generated in this manuscript are submitted with the main document and its Supplementary material uploaded to GSA figshare: https://doi.org/10.25387/g3.21623121.
