# Supplementary material for: No evidence for sex chromosomes in natural populations of the cichlid fish Astatotilapia burtoni
Source: G3 (Bethesda). 2023 Jan 17;13(3):jkad011. doi: 10.1093/g3journal/jkad011 (PMC9997565; doi:10.1093/g3journal/jkad011)
Supplement: jkad011_Supplementary_Data [file jkad011_supplementary_data.zip › File_S1.pdf]

Supplementary Material File S1 for "No evidence for sex chromosomes in natural populations of the cichlid fish *Astatotilapia burtoni*"

This file contains Supplementary Figures S1-S9 and explanations to Supplementary Tables S1-S5 (provided as separate excel files) and Files S2-S4 (provided as separate compressed files).

Supplementary Figures Legends

Figure S1. (Top) Intersex Fst values for each of the populations sampled. Blue dotted line represents the value indicative of a heterogametic system with all individuals of one sex being heterozygous and all individuals of the other sex being homozygous. Note absence of a chromosomal region associated to sex differences. (Bottom) Nucleotide diversity (pi) difference between sexes for each population. Population abbreviations correspond to Fig. 1. Points are SNPs detected in each chromosome, which are highlighted with interchanging grey and black color referring to chromosomes. Ruzizi stream (Ru1) population shows a deviation due to a lower number of female replicates.

Figure S2. Percentage of reads mapped for each one of the *A. burtoni* natural populations assemblies generated. Labels in the x-axis denote the population and the sex of the assembly (F = female, M = male), followed by the sex of samples mapped to each respective assembly and color coded with blue for males and red for females. Comparisons within each assembly and sex are showed on top. Symbols indicating statistical significance are *: $p \le 0.05$; **: $p \le 0.01$, ***: $p \le 0.001$, ****: $p \le 0.0001$, ns: $p > 0.05$. Wilcoxon rank sum post-hoc test only significant between samples in the Chitili River (Ch1) female assembly but the *p*-value computed is marginal (Benjamini-Hochberg adjusted p-value = 0.0505).

Figure S3. Sex-specific scaffolds containing regions of at least 500bp of zero read mapping coverage in the opposite sex of the assembled genome. (A) Number of sex-specific scaffolds assigned to each chromosome or not placed regions in the reference genome of the Nile tilapia. Left, numbers for female assemblies. Right, numbers for male assemblies. (B) Number of sex-specific scaffolds assigned to each chromosome visualized per population. Left, for female assemblies. Right, for male assemblies. (C) Number of BLAST entries with similar sequence name description for each scaffold. Left, female assemblies, right, male assemblies.

Figure S4. Coverage of female (x-axis) and male (y-axis) k-mers in *A. burtoni* natural populations. Numbers of k-mers detected in each category per population are shown. Different colors indicate different k-mer categories: dark blue = Y-k-mers = male-specific; light blue Z-k-mers = male-biased; red = X-k-mers = female-biased; and orange = W-k-mers = female-specific. Population abbreviations correspond to those in Fig. 1. Kmer categories for illustrative purposes highlighted in RuL population.

Figure S5. Intersection plots for X-, Z- and W-k-mers for all *A. burtoni* natural populations. Note that Y-k-mers are depicted in Fig. 5. Blue bars indicate the number of corresponding k-mers for each population. We did not find a single k-mer shared across all populations in any category.

Figure S6. Main intersections plotted for all k-mers found in the different categories intersected with all categories. Blue bars depict the number of corresponding k-mers for each population and category. We did not find a single k-mer shared across all populations in the same category but some k-mers are found either in the opposite category (i.e., Y in W) in other populations or also in closely related populations but in the sex-biased category (i.e., Y and X).

Figure S7. (A) Phylogenetic tree for all specimens using AAF with a k-mer length of 25 bp. Red color is used to indicate female specimens, blue color for males. (B) Phylogenetic tree for all specimens using AAF with a k-mer length of 31 bp, red color is used to indicate female specimens, blue color for males. Population abbreviations correspond to Fig. 1.

Figure S8. Left - phylogenetic trees generated with AAF with a k-mer length of 25 bp. Right - phylogenetic trees generated with AAF with a k-mer length of 31 bp. ed color is used to indicate female specimens, blue color for males. Population abbreviations correspond to Fig. 1.

Figure S9. Phylogenetic trees generated with AAF with a k-mer length of 25 bp for each chromosome. Note that in the previously detected sex chromosomes (i.e., LG14-05, LG13, LG18) individual samples are not clustering per sex a. Chromosome names are located on top. Red color is used to indicate female specimens, blue color for males. Population abbreviations correspond to Fig. 1.

Supplementary Tables

Table S1. BLAST hit scores of previously identified male-associated markers against the *de novo A. burtoni* assemblies. Each spreadsheet contains information for each one of the two previously detected male-associated RAD-sequencing markers.

Table S2. Genome assembly qualities for each population and sex.

Table S3. Back-mapping statistics for all *A. burtoni de novo* assemblies.

Table S4. Genome-wide coverage in 10Kb windows for female (spreadsheets ending in _F) and male (spreadsheets ending in _M) assemblies. Windows surpassing the top 99% quantile of the median-normalized coverage ratio between sexes, showing the median-normalized coverage difference between sexes above 0.25 in the sex of the corresponding assembly. Within each window, median values for each position were used to compute Wilcoxon post-hoc tests. Each spreadsheet shows the depicted windows per population.

Table S5. Statistics for the number of significant 10Kb windows found per chromosome in each one of the *de novo* assemblies generated. For each chromosome the associated one-sided Fisher's exact test of the number of significant windows per each population and sex-associated *p*-value, 95% confidence interval, odds ratio, and Bonferroni adjusted *p*-value are shown.

Table S6. Sequence characterization of scaffolds with sex-specific sequences. Corresponding regions of scaffolds with sex-specific sequences in the Nile tilapia reference genome, and sequence description name obtained from the corresponding protein-coding sequence are indicated. Highlighted in light yellow are chromosomes with more than one hit. Selected regions for visualization on the chromosome with the longest alignment score.

File S2

This file contains large figures representing heatmaps with sample clustering of individual genotypes at SNPs located in the the previously described LG05, LG18 and LG13 sex-determining region in *A. burtoni* (Böhne et al. 2016; Roberts et al. 2016). Genotypes in blue are heterozygous, in black homozygous reference, and in grey homozygous alternative.

HeatmapXYRegionLG05HetBlueNaWhite.pdf

Heatmap clustering for genotypes on the previously described XY sex-determining region on LG05.

HeatmapXYRegionLG18HetBlueNaWhite.pdf

Heatmap clustering for genotypes on the previously described XY sex-determining region on LG18.

HeatmapZWRegionHetBlueNaWhite.pdf

Heatmap clustering for genotypes on the previously described ZW sex-determining region on LG13.

File S3

This file contains large figures investigating sex differences in genome coverage.

Female_Assemblies.pdf

Coverage 10Kb window visualization for each population and chromosome for female assemblies. Each page shows the mapping coverage for each chromosome within a population. The figures are sorted accordingly to chromosome numbers (columns) first showing the four identified sex chromosomes in *A. burtoni* LG05/ 14, LG13 and LG18, and then numerically sorted; rows correspond to species. X-axis: chromosomal positions; y-axis: log2 normalized-median coverage. Red lines and dots = female coverage, blue lines and dots = male coverage. yellow lines = windows in the upper 99% quantile of male-female coverage ratio and with normalized-median coverage above 0.25 in females.

Male_Assemblies.pdf

Coverage 10Kb window visualization for each population and chromosome for male assemblies. Each page shows the mapping coverage for each chromosome within a population. The figures are sorted accordingly to chromosome numbers (columns) first showing the four identified sex chromosomes in *A. burtoni* LG05/ 14, LG13 and LG18, and then numerically sorted; rows correspond to species. X-axis: chromosomal positions; y-axis: log2 normalized-median coverage. Red lines and dots = female coverage, blue lines and dots = male coverage. yellow lines = windows in the upper 99% quantile of male-female coverage ratio and with normalized-median coverage above 0.25 in males.

File S4

This file contains large figures representing sex-specific genome sequence analyses.

Female-specific_regions_in_Scaffolds.pdf

Scaffolds with female-specific regions of at least 500bp length. Each separate sheet shows the scaffolds found in each population. The right-hand side labels show the assigned scaffold name. Red = female median coverage normalized by overall median depth for the corresponding population across the *de novo* assembled genome, blue = male median coverage normalized by overall median depth for the corresponding population across the *de novo* assembled genome.

Male-specific_regions_in_Scaffolds.pdf

Scaffolds with male-specific regions of at least 500bp length. Each separate sheet shows the scaffolds found in each population. The right-hand side labels show the assigned scaffold name. Red = female median coverage normalized by overall median depth for the corresponding population across the *de novo* assembled genome, blue = male median coverage normalized by overall median depth for the corresponding population across the *de novo* assembled genome.

Intersex Fst across populations

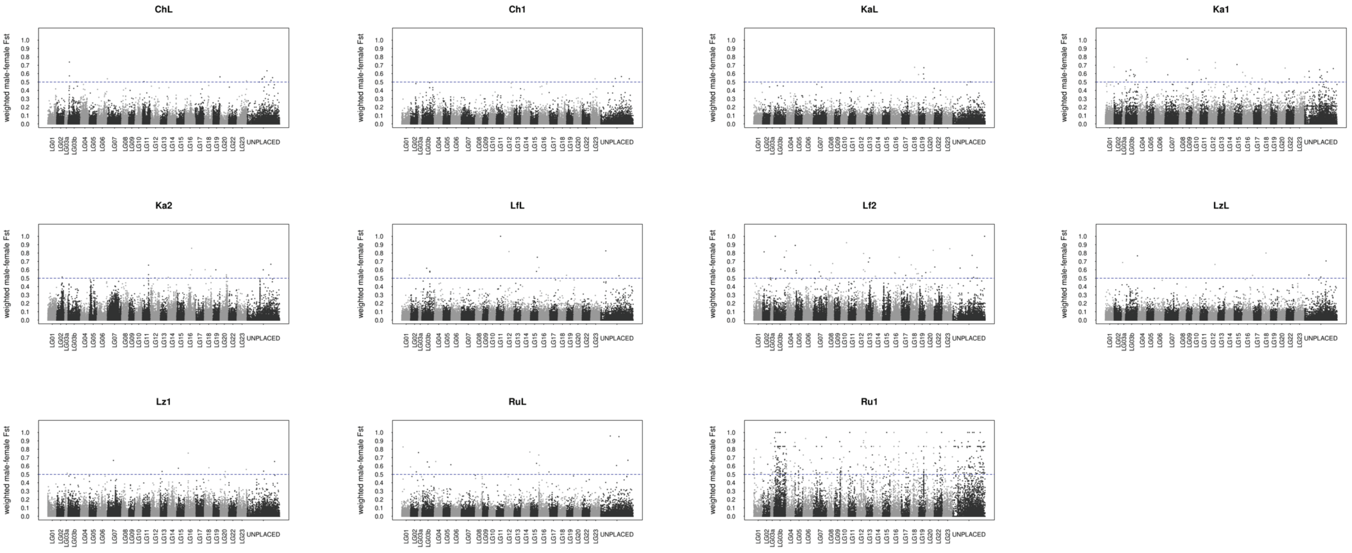

Nucleotide diversity (pi) difference across populations

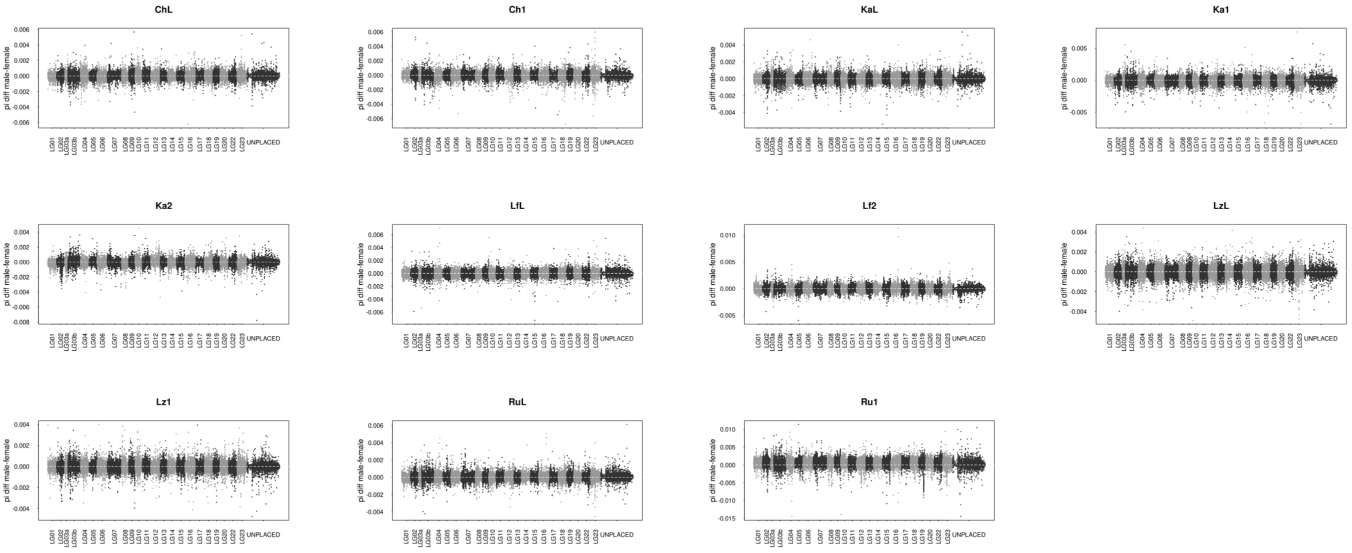

Figure S1. (Top) Intersex Fst for each of the populations sampled. Blue dotted line represents statistical threshold to consider a SNP to be significant. Note absence of a chromosomal region associated to sex differences. (Bottom) Nucleotide diversity (pi) difference between sexes for each population. Population abbreviations are as in Figure 1. Points are SNPs detected in each chromosome, which are highlighted with grey and black color depending on the chromosome. Ruzizi stream (Ru1) population shows increased patterns due to the low number of female replicates.

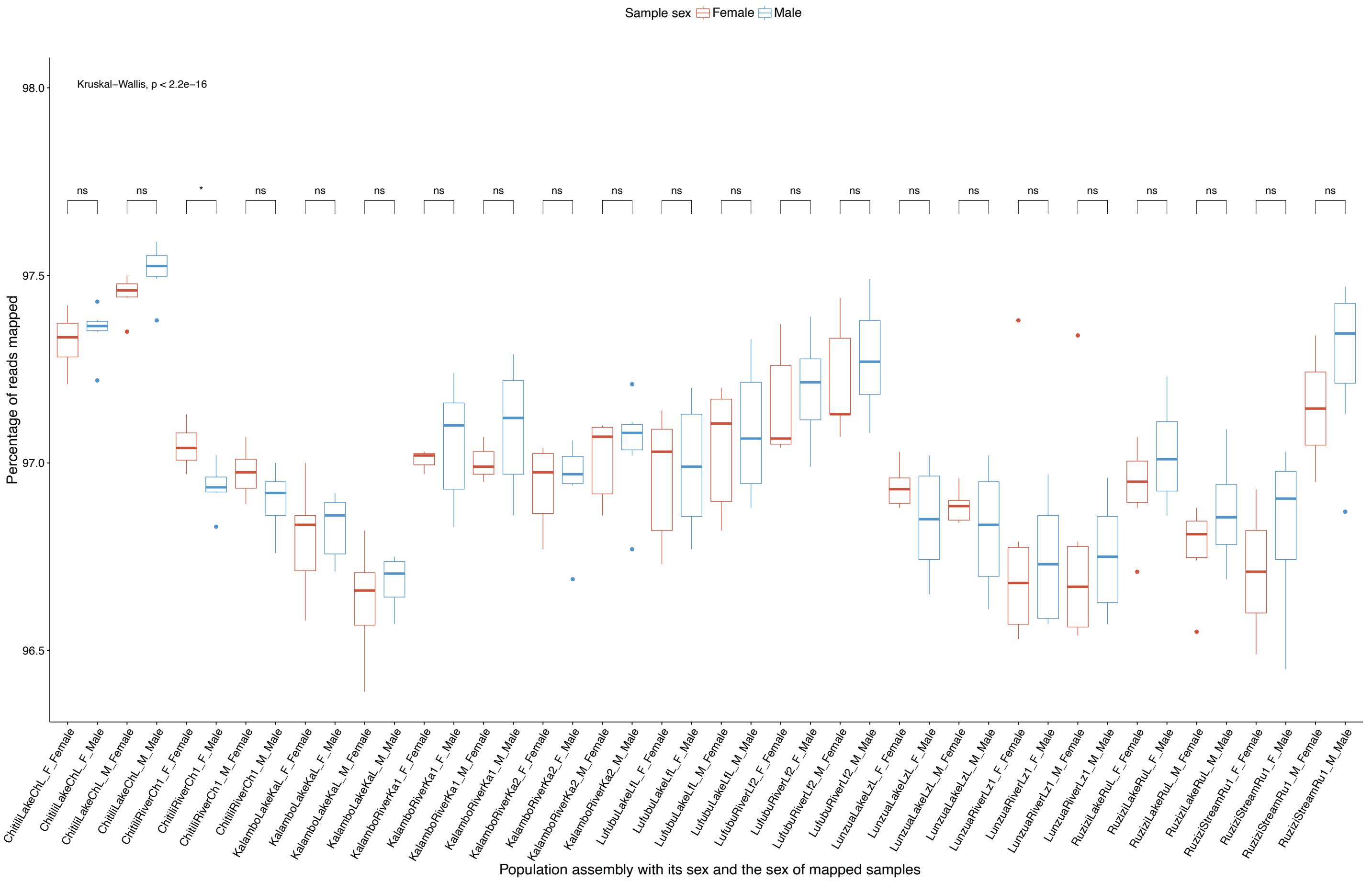

Figure S2. Percentage of reads mapped for each one of the *A. burtoni* natural populations assemblies generated. Labels in the x-axis denote the population and the sex of the assembly (F = female, M = male), followed by the sex of samples mapped to each respective assembly and color coded with blue for males and red for females. Comparisons within each assembly and sex are showed on top. Symbols indicating statistical significance are *: p <= 0.05; **: p <= 0.01, ***: p <= 0.001, ****: p <= 0.0001, ns: p > 0.05 of Wilcoxon rank sum post hoc test after Benjamini-Hochberg adjustment.

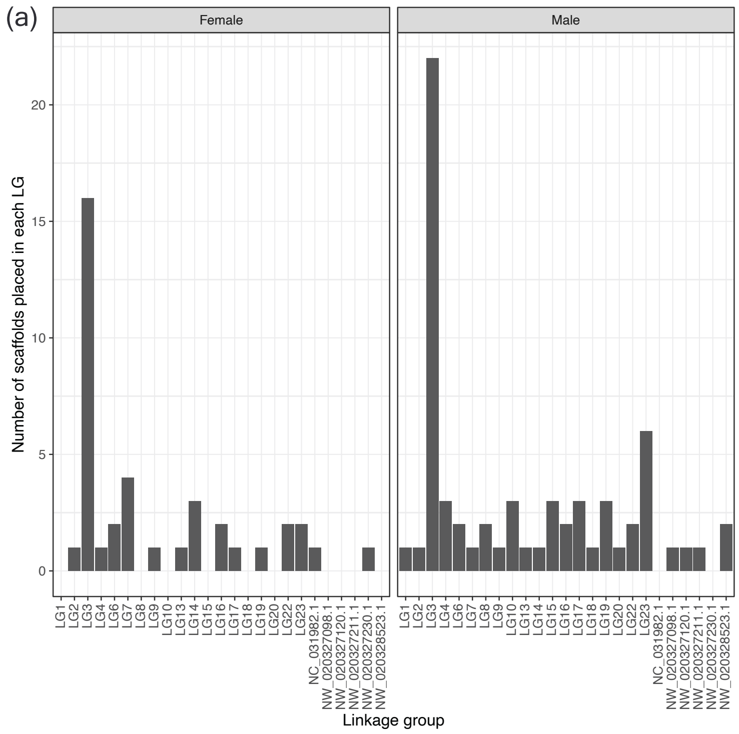

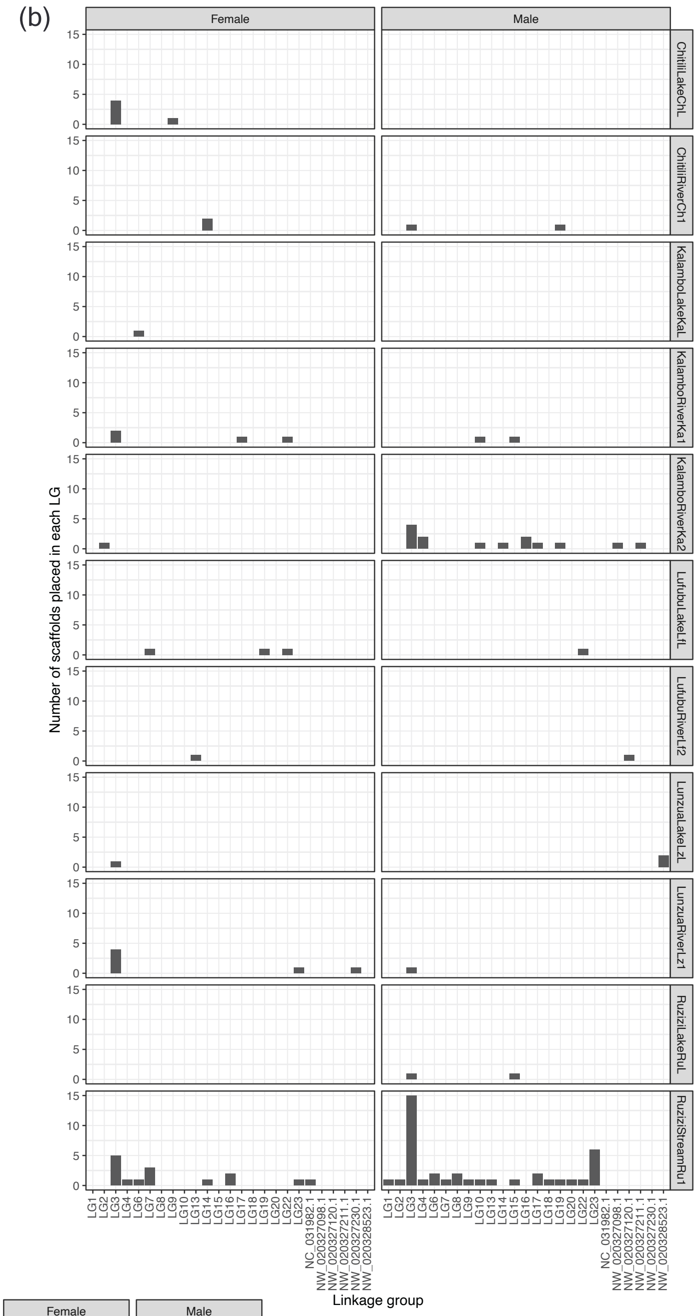

Figure S3. Sex-specific scaffolds with a region of at least 500bp of zero counts in the opposite sex of the assembled genome.

(a) Number of sex-specific scaffolds assigned to each chromosome or not-placed regions of the Nile tilapia reference genome. Left, numbers for female assemblies. Right, numbers for male assemblies.

(b) Number of sex-specific scaffolds assigned to each chromosome visualized per population. Left, for female assemblies. Right, for male assemblies.

(c) Number of BLAST entries with similar sequence name description for each scaffold. Left, female assemblies, right, male assemblies.

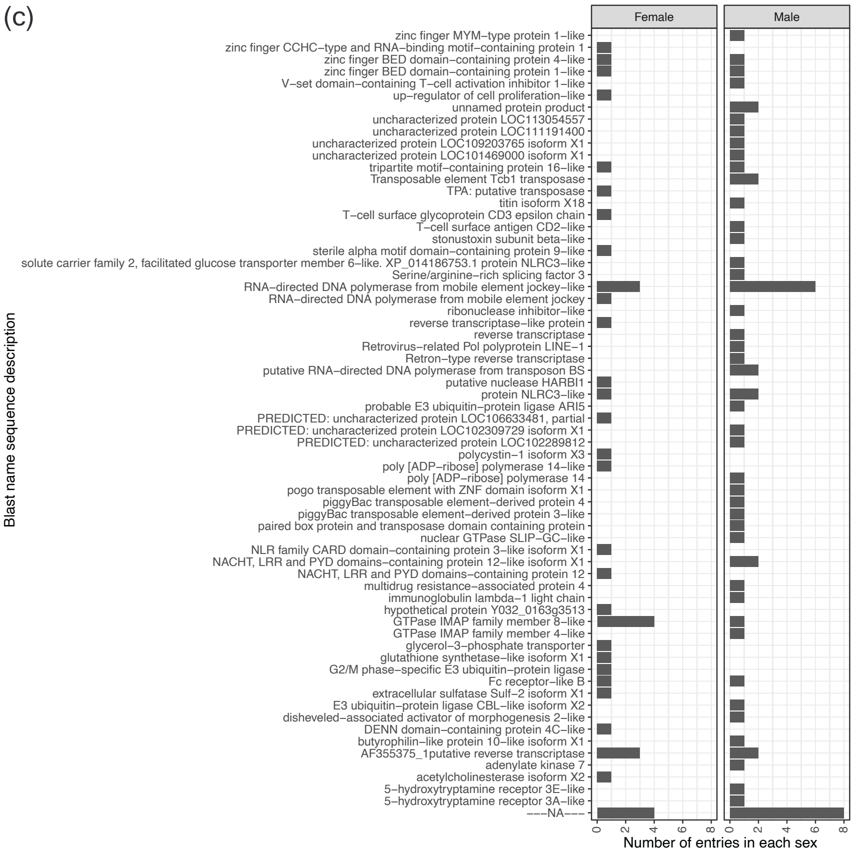

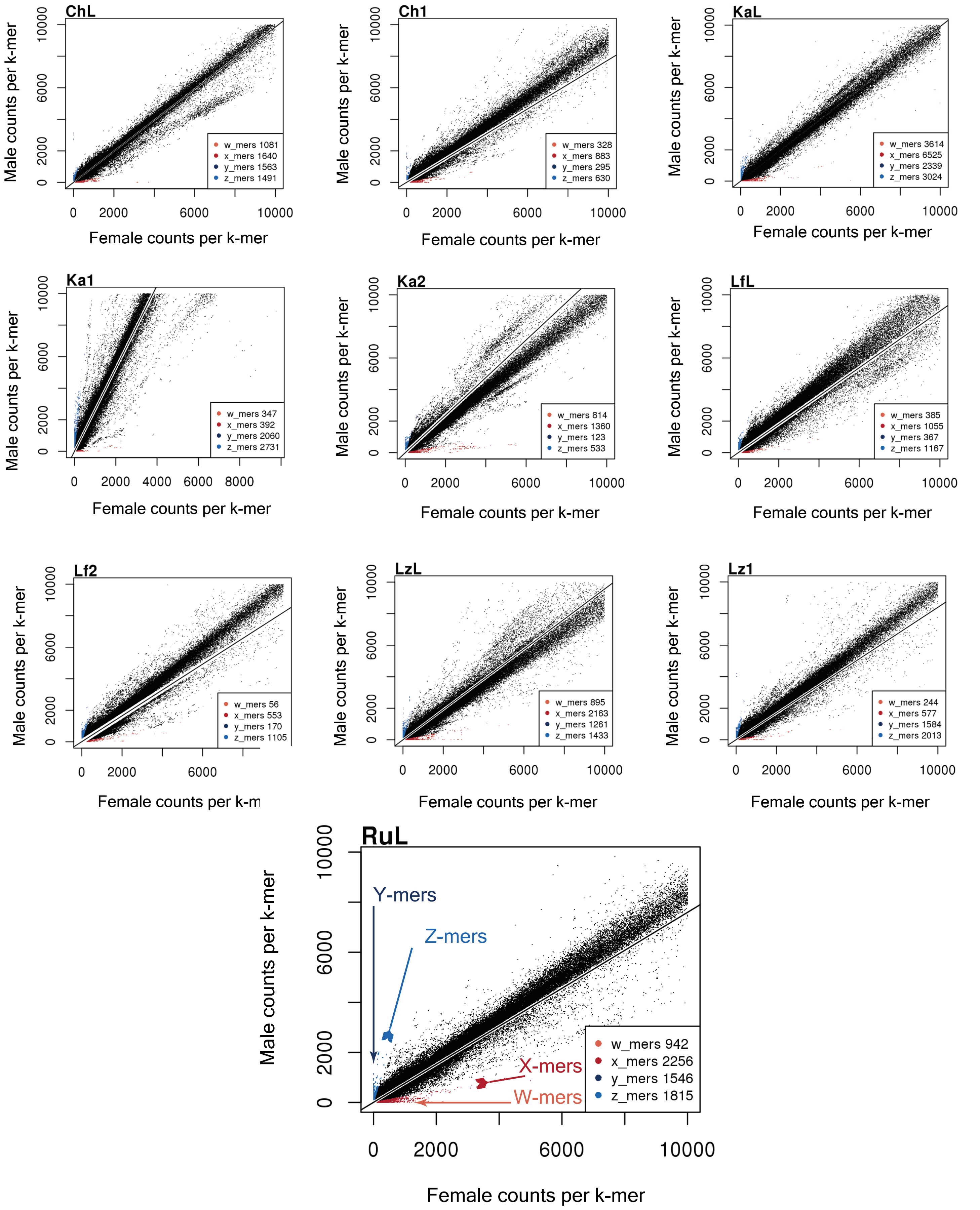

Figure S4. Coverage of female (x-axis) and male (y-axis) k-mers in *A. burtoni* natural populations. Numbers of k-mers detected in each category per population are shown. Different colors indicate different k-mer categories: dark blue = Y-k-mers = male-specific; light blue Z-k-mers = male-biased; red = X-k-mers = female-biased; and orange = W-k-mers = female-specific. Population abbreviations are as in Fig. 1. K-mer categories are examplatory highlighted in the RuL population.

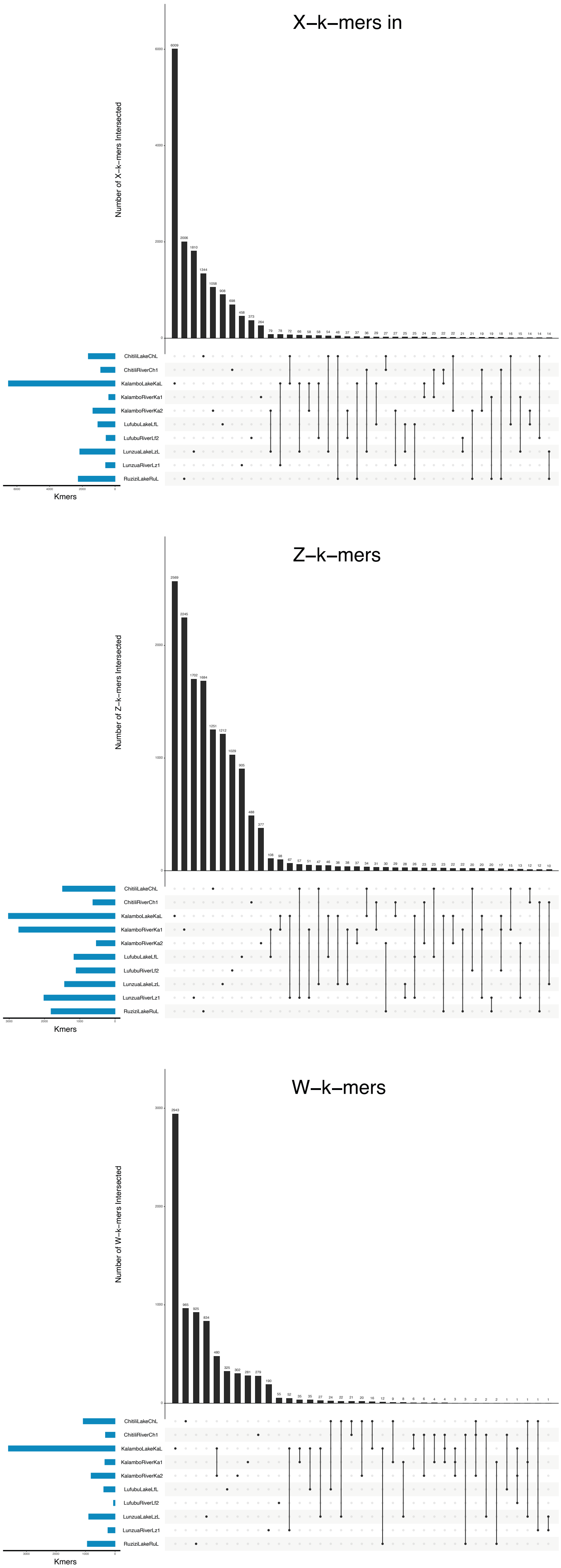

Figure S5. Intersection plots for X-, Z- and W-k-mers for all *A. burtoni* natural populations. Note that Y-k-mers are in the main text under Figure 5. Blue bars are depicting the number of corresponding k-mers for each population. We did not find a single k-mer shared across all populations in any category.

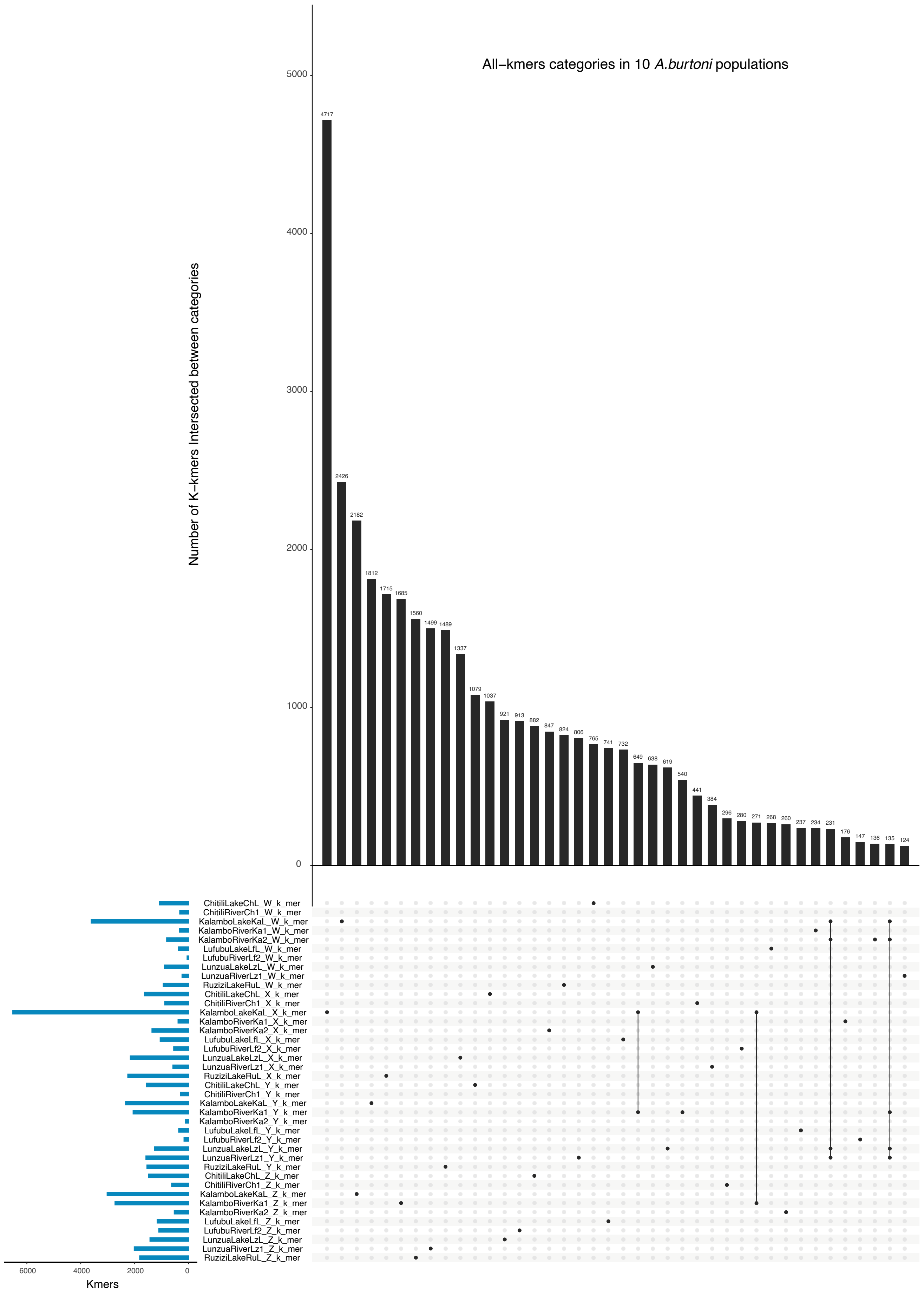

Figure S6. Main intersections plotted for all the k-mers found between different categories. Blue bars depict the number of corresponding k-mers for each population and category. We did not find a single k-mer shared across all populations in and all categories but some k-mers are found either in the opposite category (i.e., Y in W) in other populations or also in closely related populations but in the sex-biased category (i.e., Y and X).

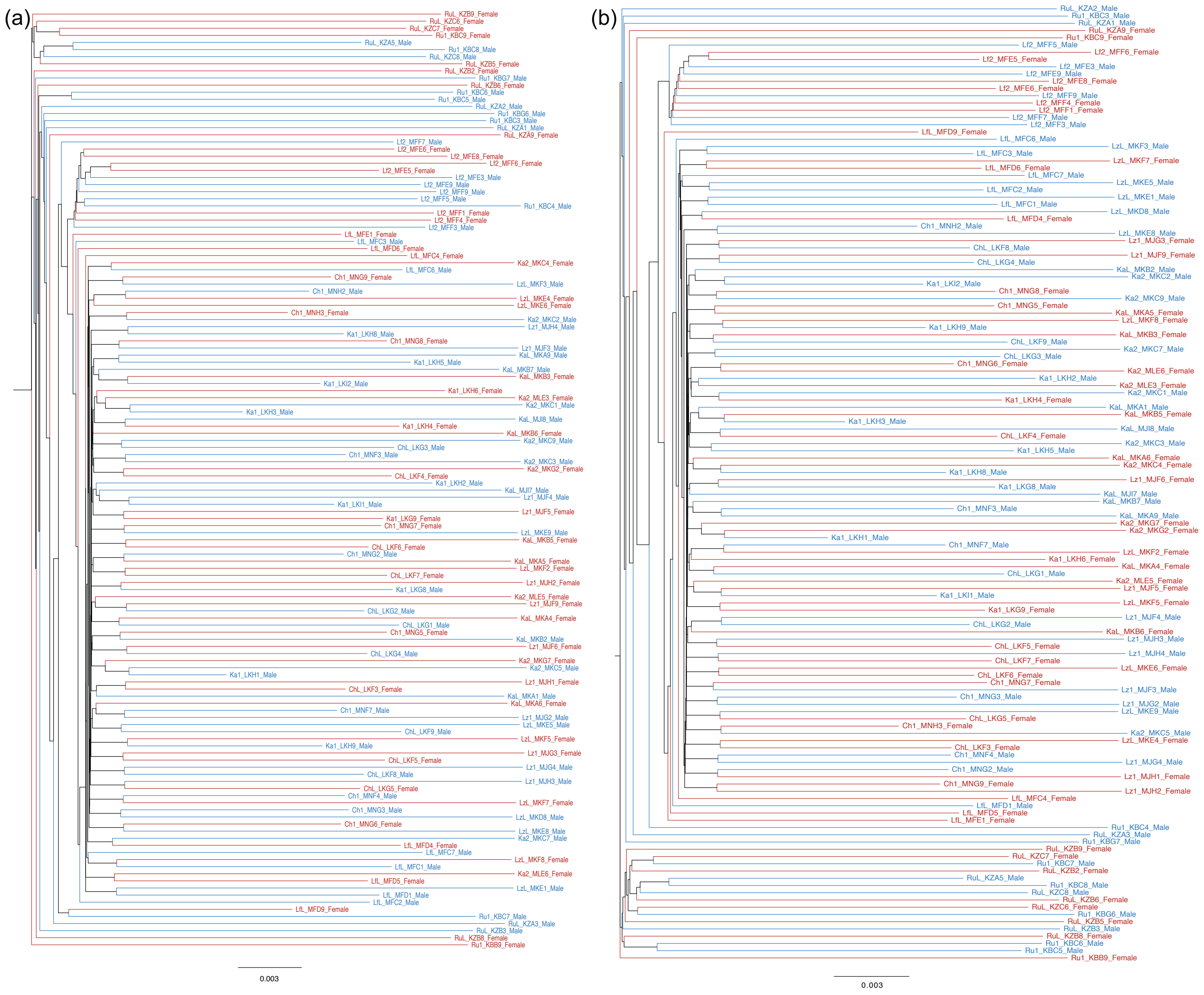

Figure S7. a) Phylogenetic tree for all specimens using AAF with a k-mer length of 25 bp, red color is use to show female specimens and blue color for males. b) Phylogenetic tree for all specimens using AAF with a k-mer length of 31 bp, red color is use to show female specimens and blue color for males. Population abbreviations are as Fig. 1.

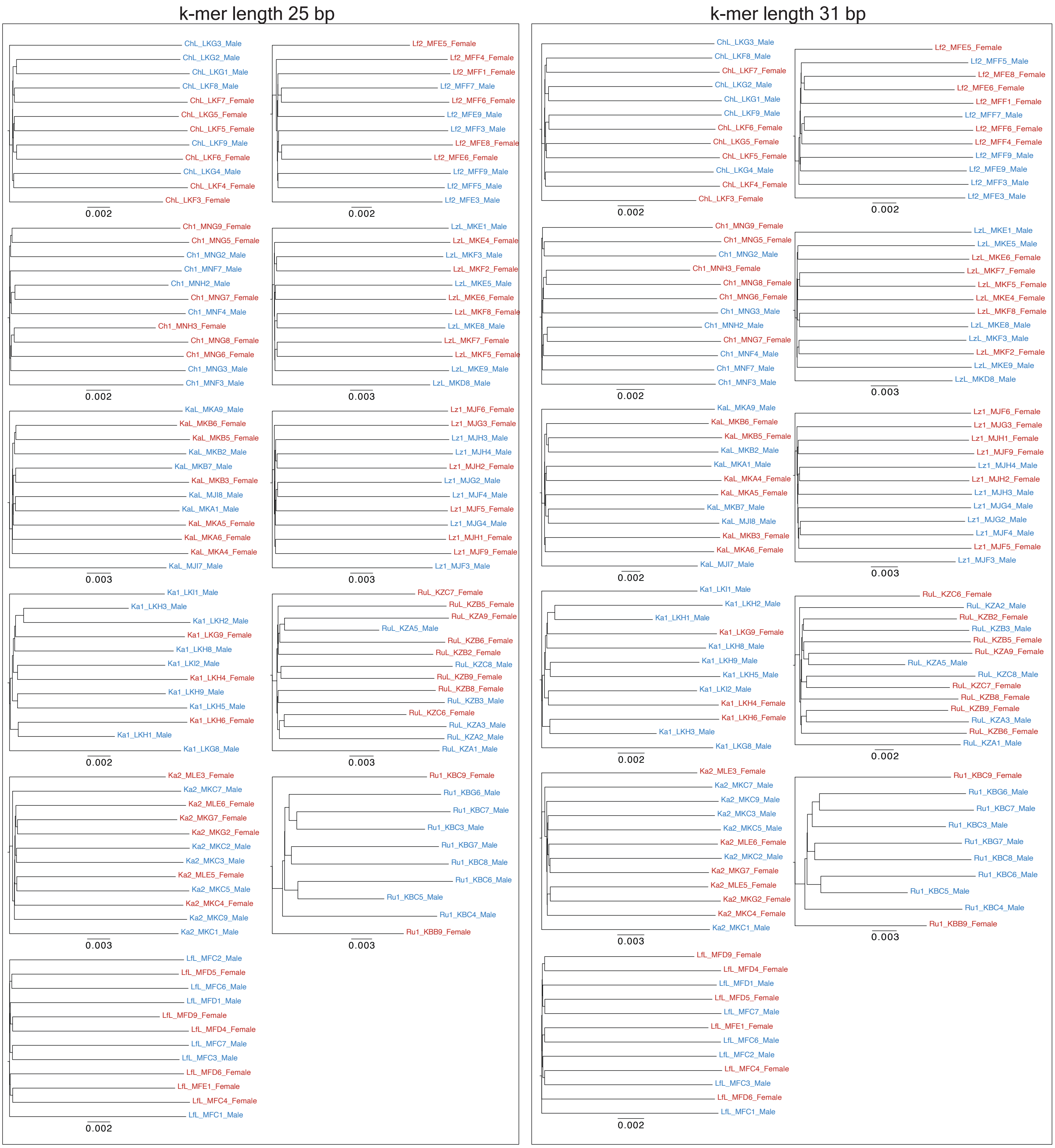

Figure S8. Left - phylogenetic trees generated with AAF with a k-mer length of 25 bp. Right - phylogenetic trees generated with AAF with a k-mer length of 31 bp. Red color is use to show female specimens and blue color for males. Population abbreviations are as in Fig. 1.

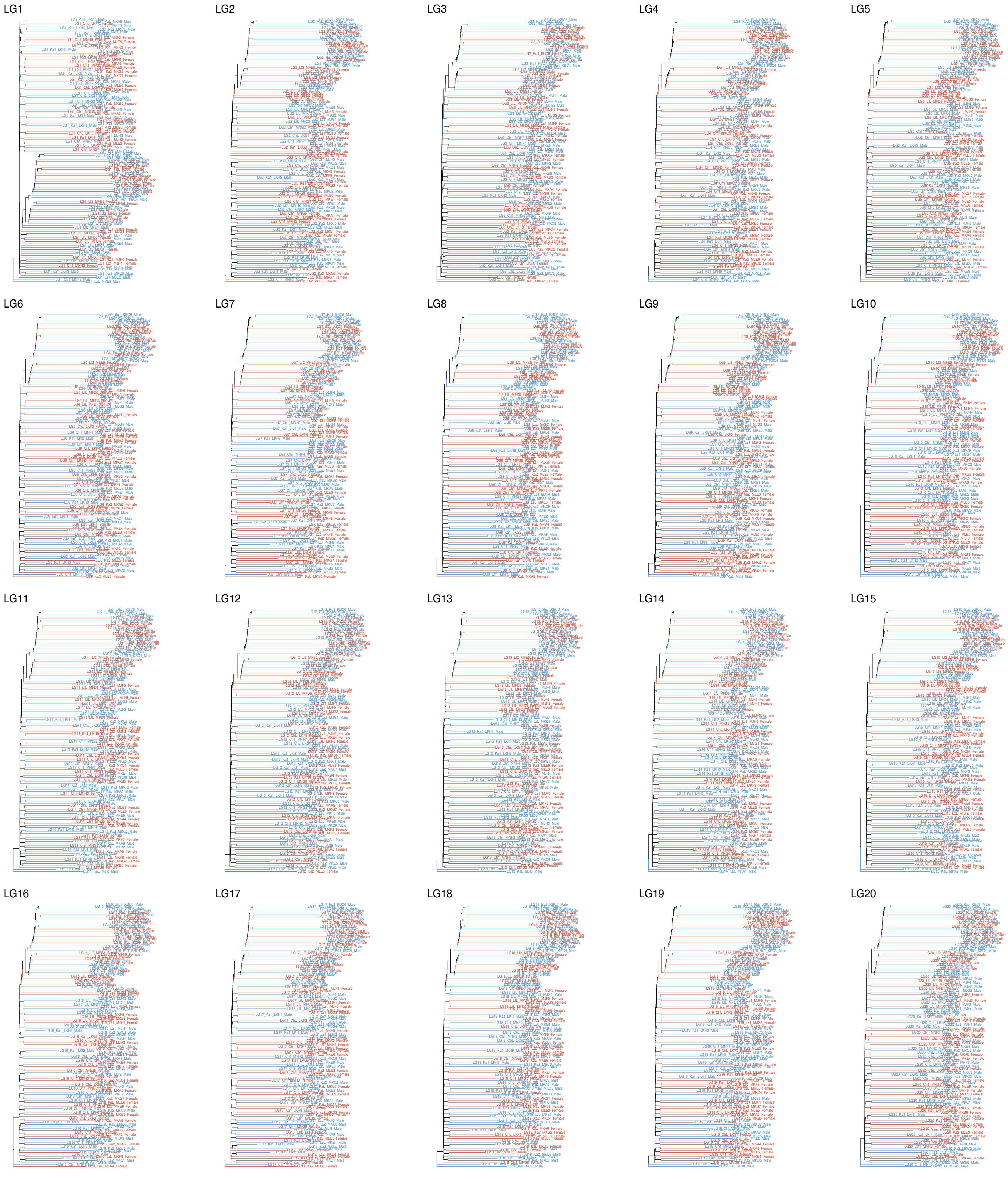

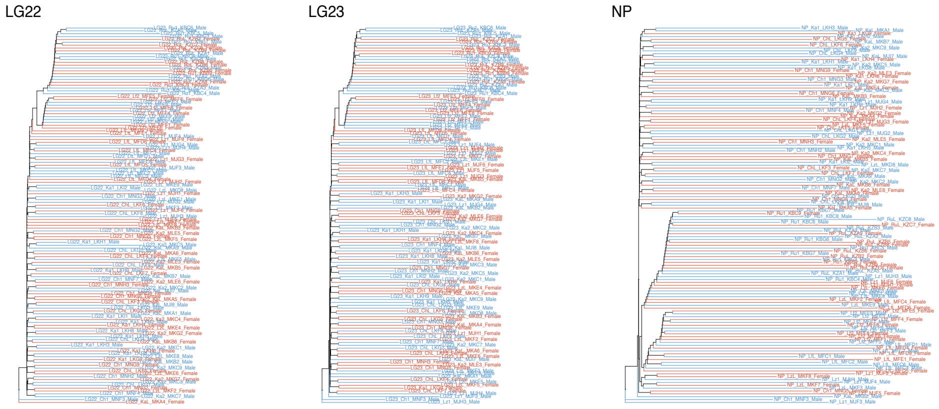

Figure S9. Phylogenetic trees generated with AAF with a k-mer length of 25 bp for each chromosome. Note that in the previously detected sex chromosomes (i.e., LG14-05, LG13, LG18) individual samples are not clustering per sex as it was expected. Chromosome names are located on top. Red color is use to show female specimens and blue color for males. Population abbreviations are as in Fig. 1.
