# Supplementary material for: No evidence for sex chromosomes in natural populations of the cichlid fish Astatotilapia burtoni
Source: G3 (Bethesda). 2023 Jan 17;13(3):jkad011. doi: 10.1093/g3journal/jkad011 (PMC9997565; doi:10.1093/g3journal/jkad011)
Supplement: jkad011_Supplementary_Data [file jkad011_supplementary_data.zip › File_S2.zip!/File_S1/HeatmapXYRegionLG05HetBlueNaWhite.pdf]

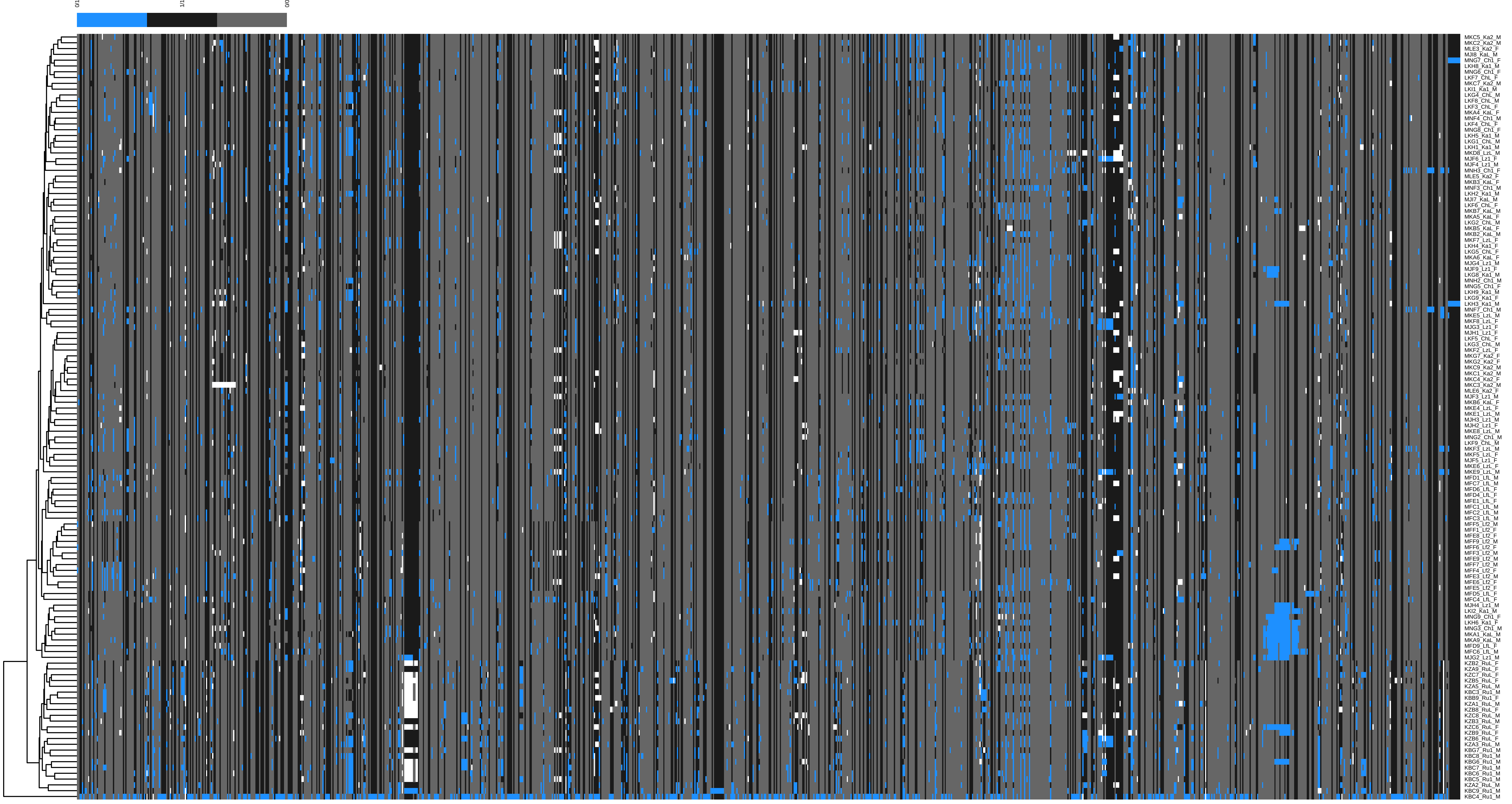
0/1
1/1
0/0
MKC5_Ka2_M
MKC2_Ka2_M
MLE3_Ka2_F
MJI8_KaL_M
MNG7_Ch1_F
LKH8_Ka1_M
MNG6_Ch1_F
LKF7_ChL_F
MKC7_Ka2_M
LKI1_Ka1_M
LKG4_ChL_M
LKF8_ChL_M
LKF3_ChL_F
MKA4_KaL_F
MNF4_Ch1_M
LKF4_ChL_F
MNG8_Ch1_F
LKH5_Ka1_M
LKG1_ChL_M
LKH1_Ka1_M
MKD8_LzL_M
MJF6_Lz1_F
MJF4_Lz1_M
MNH3_Ch1_F
MLE5_Ka2_F
MKB3_KaL_F
MNF3_Ch1_M
LKH2_Ka1_M
MJI7_KaL_M
LKF6_ChL_F
MKB7_KaL_M
MKA5_KaL_F
LKG2_ChL_M
MKB5_KaL_F
MKB2_KaL_M
MKF7_LzL_F
LKH4_Ka1_F
LKG5_ChL_F
MKA6_KaL_F
MJG4_Lz1_M
MJF9_Lz1_F
LKG8_Ka1_M
MNH2_Ch1_M
MNG5_Ch1_F
LKH9_Ka1_M
LKG9_Ka1_F
LKH3_Ka1_M
MNF7_Ch1_M
MKE5_LzL_M
MKF8_LzL_F
MJG3_Lz1_F
MJH1_Lz1_F
LKF5_ChL_F
LKG3_ChL_M
MKF2_LzL_F
MKG7_Ka2_F
MKG2_Ka2_F
MKC9_Ka2_M
MKC1_Ka2_M
MKC4_Ka2_F
MKC3_Ka2_M
MLE6_Ka2_F
MJF3_Lz1_M
MKB6_KaL_F
MKE4_LzL_F
MKE1_LzL_M
MJH3_Lz1_M
MJH2_Lz1_F
MKE8_LzL_M
MNG2_Ch1_M
LKF9_ChL_M
MKF3_LzL_M
MKF5_LzL_F
MJF5_Lz1_F
MKE6_LzL_F
MKE9_LzL_M
MFD1_LfL_M
MFC7_LfL_M
MFD6_LfL_F
MFD4_LfL_F
MFE1_LfL_F
MFC1_LfL_M
MFC2_LfL_M
MFC3_LfL_M
MFF5_Lf2_M
MFF1_Lf2_F
MFE8_Lf2_F
MFF9_Lf2_M
MFF6_Lf2_F
MFF3_Lf2_M
MFE9_Lf2_M
MFF7_Lf2_M
MFF4_Lf2_F
MFE3_Lf2_M
MFE6_Lf2_F
MFE5_Lf2_F
MFD5_LfL_F
MFC4_LfL_F
MJH4_Lz1_M
LKI2_Ka1_M
MNG9_Ch1_F
LKH6_Ka1_F
MNG3_Ch1_M
MKA1_KaL_M
MKA9_KaL_M
MFD9_LfL_F
MFC6_LfL_M
MJG2_Lz1_M
KZB2_RuL_F
KZA9_RuL_F
KZC7_RuL_F
KZB5_RuL_F
KZA5_RuL_M
KBC3_Ru1_M
KBB9_Ru1_F
KZA1_RuL_M
KZB8_RuL_F
KZC8_RuL_M
KZB3_RuL_M
KZC6_RuL_F
KZB9_RuL_F
KZB6_RuL_F
KZA3_RuL_M
KBG7_Ru1_M
KBC8_Ru1_M
KBG6_Ru1_M
KBC7_Ru1_M
KBC6_Ru1_M
KBC5_Ru1_M
KZA2_RuL_M
KBC9_Ru1_M
KBC4_Ru1_M
