# Supplementary material for: No evidence for sex chromosomes in natural populations of the cichlid fish Astatotilapia burtoni
Source: G3 (Bethesda). 2023 Jan 17;13(3):jkad011. doi: 10.1093/g3journal/jkad011 (PMC9997565; doi:10.1093/g3journal/jkad011)
Supplement: jkad011_Supplementary_Data [file jkad011_supplementary_data.zip › File_S2.zip!/File_S1/File S1_README.rtf]

File S1
Heatmap clustering for genotypes on the previously described LG05, LG18 and LG13 sex-determining region in A. burtoni (Böhne et al. 2016; Roberts et al. 2016). Genotypes in blue are heterozygous, in black reference homozygous, and in grey alternative homozygous.

HeatmapXYRegionLG05HetBlueNaWhite.pdf
Heatmap clustering for genotypes on the previously described XY sex-determining region LG05.
HeatmapXYRegionLG18HetBlueNaWhite.pdf
Heatmap clustering for genotypes on the previously described XY sex-determining region LG18.
HeatmapZWRegionHetBlueNaWhite.png
Heatmap clustering for genotypes on the previously described ZW sex-determining region LG13.
