# Supplementary material for: No evidence for sex chromosomes in natural populations of the cichlid fish Astatotilapia burtoni
Source: G3 (Bethesda). 2023 Jan 17;13(3):jkad011. doi: 10.1093/g3journal/jkad011 (PMC9997565; doi:10.1093/g3journal/jkad011)

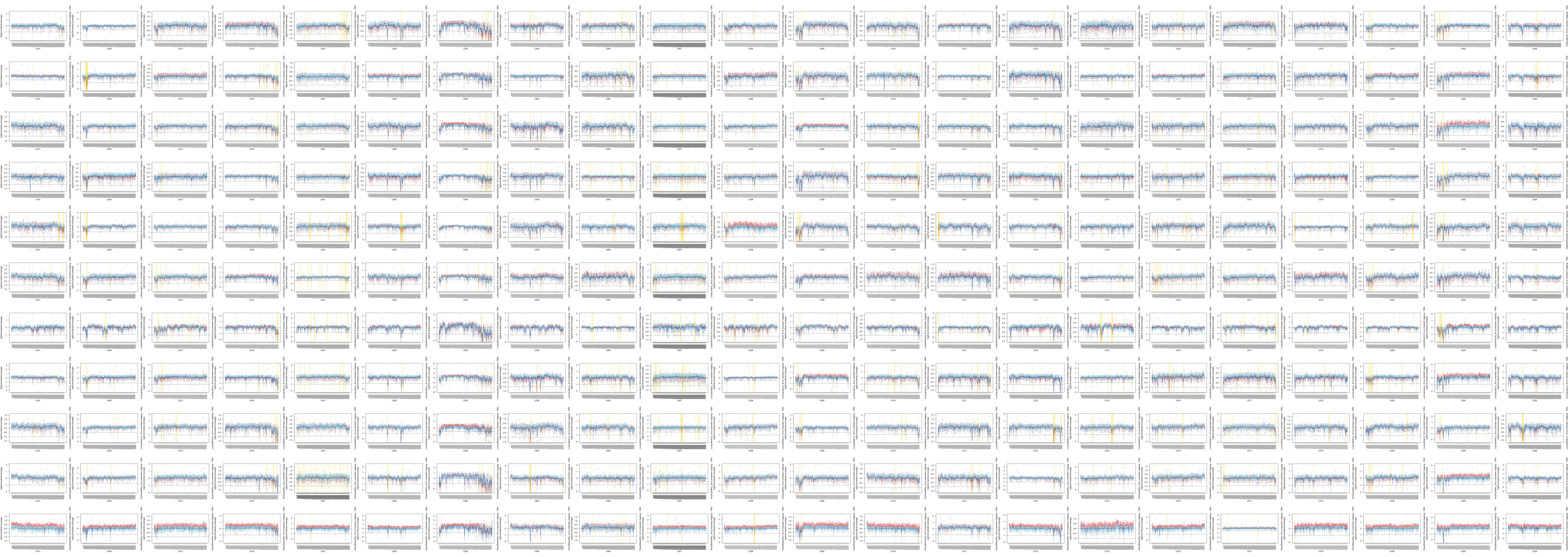

Supplement: jkad011_Supplementary_Data [file jkad011_supplementary_data.zip › File_S3.zip!/Male_Assemblies.pdf]
