# Supplementary material for: No evidence for sex chromosomes in natural populations of the cichlid fish Astatotilapia burtoni
Source: G3 (Bethesda). 2023 Jan 17;13(3):jkad011. doi: 10.1093/g3journal/jkad011 (PMC9997565; doi:10.1093/g3journal/jkad011)
Supplement: jkad011_Supplementary_Data [file jkad011_supplementary_data.zip › File_S3.zip!/File_S2_README.rtf]

File S2
Female_Assemblies file
Coverage 10Kb window visualization for each population and chromosome for female assemblies. Each page shows the mapping coverage for each chromosome within a population. The figures are sorted accordingly to chromosome numbers (columns) first showing the four identified sex chromosomes in A. burtoni LG5- LG14, LG13 and LG18, and then numerically sorted, and each row is a species. X-axis is showing genomic positions; y-axis shows the log_2_ normalized-median coverage. Red lines and dots = females, blue lines and dots = males. Highlighted in yellow lines are windows in the upper 99% quantile of male-female ratio and with normalized-median coverage above 0.25 in females.
Male_Assemblies file
Coverage 10Kb window visualization for each population and chromosome for male assemblies. Each page shows the mapping coverage for each chromosome within a population. The figures are sorted accordingly to chromosome numbers (columns) first showing the four identified sex chromosomes in A. burtoni LG5- LG14, LG13 and LG18, and then numerically sorted, and each row is a species. X-axis is showing genomic positions; y-axis shows the log_2_ normalized-median coverage. Red lines and dots = females, blue lines and dots = males. Highlighted in yellow lines are windows in the upper 99% quantile of female-male ratio and with normalized-median coverage above 0.25 in males.
