# Supplementary material for: No evidence for sex chromosomes in natural populations of the cichlid fish Astatotilapia burtoni
Source: G3 (Bethesda). 2023 Jan 17;13(3):jkad011. doi: 10.1093/g3journal/jkad011 (PMC9997565; doi:10.1093/g3journal/jkad011)

Regions with zero coverage bigger than 500 bp in males for female assemblies

ChitiliLakeChL_F

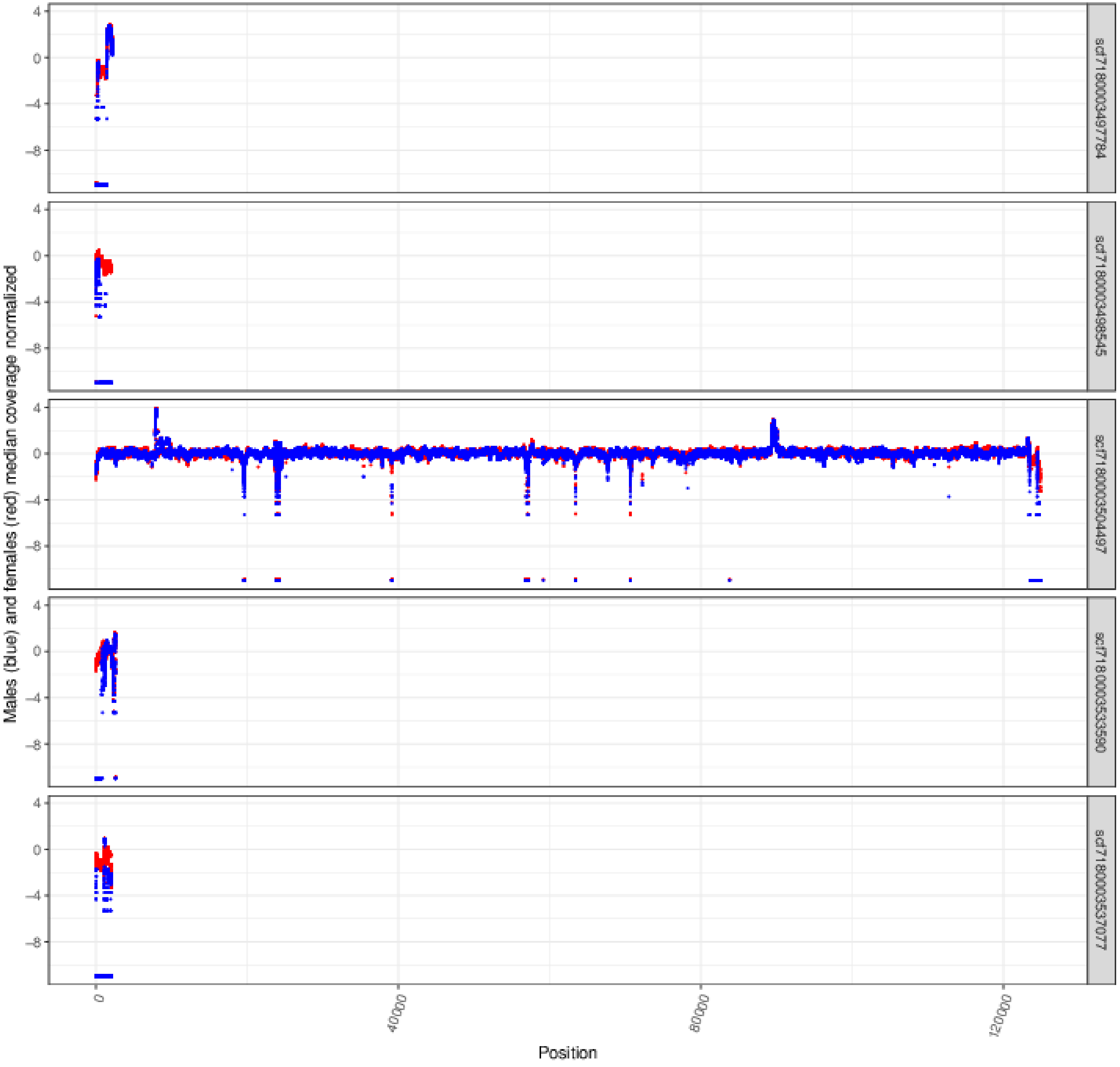

Regions with zero coverage bigger than 500 bp in males for female assemblies

ChitiliRiverCh1_F

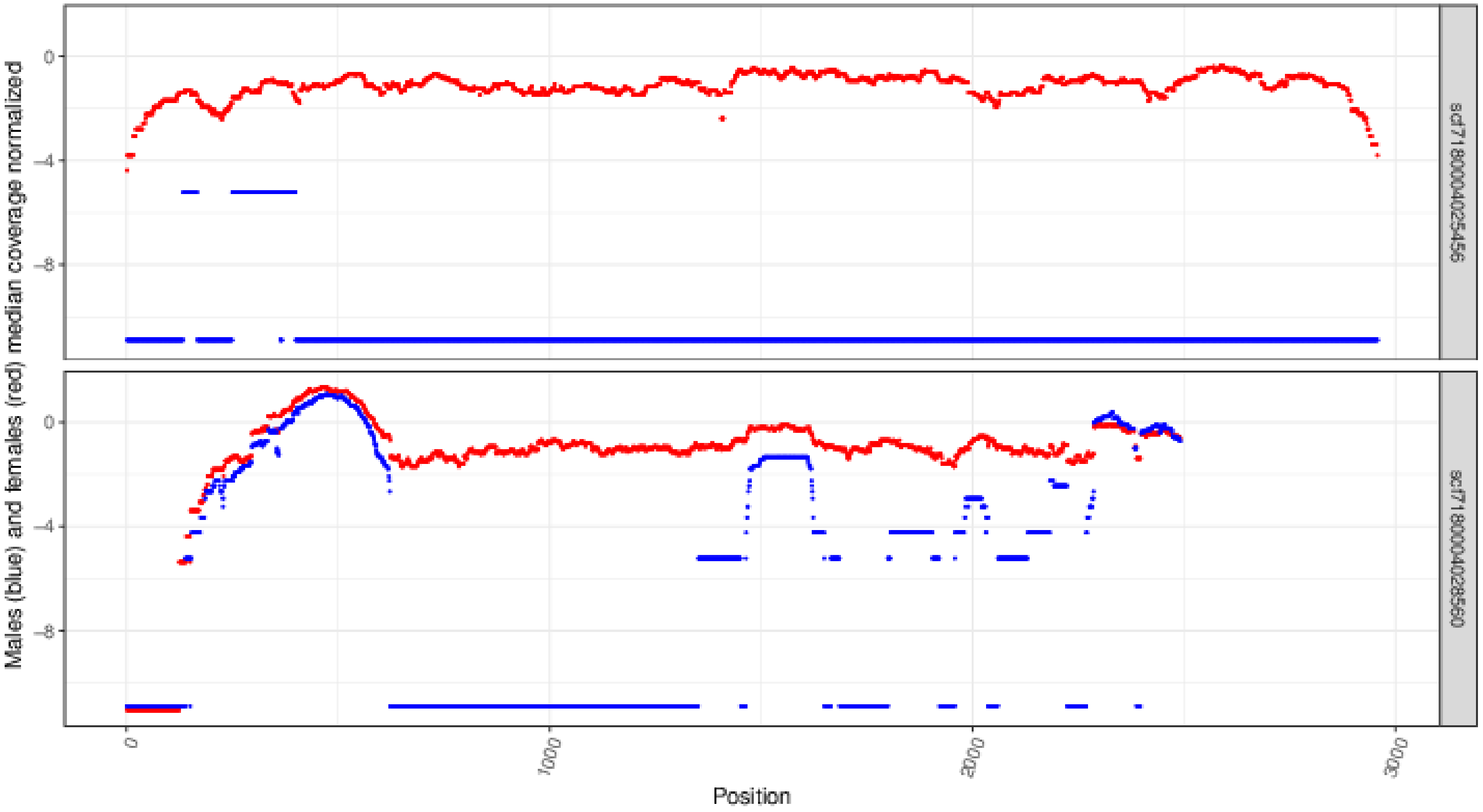

Regions with zero coverage bigger than 500 bp in males for female assemblies

KalamboLakeKaL_F

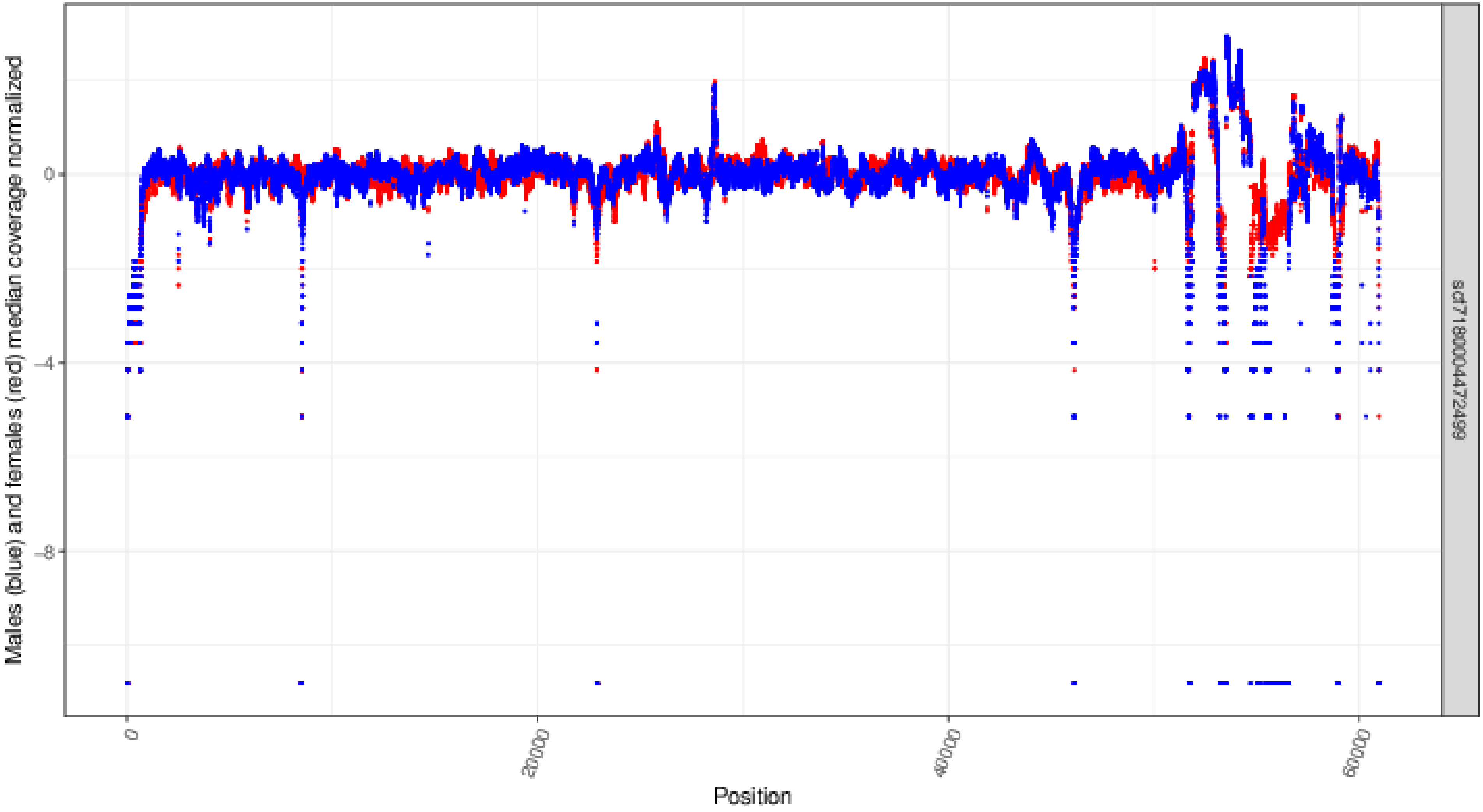

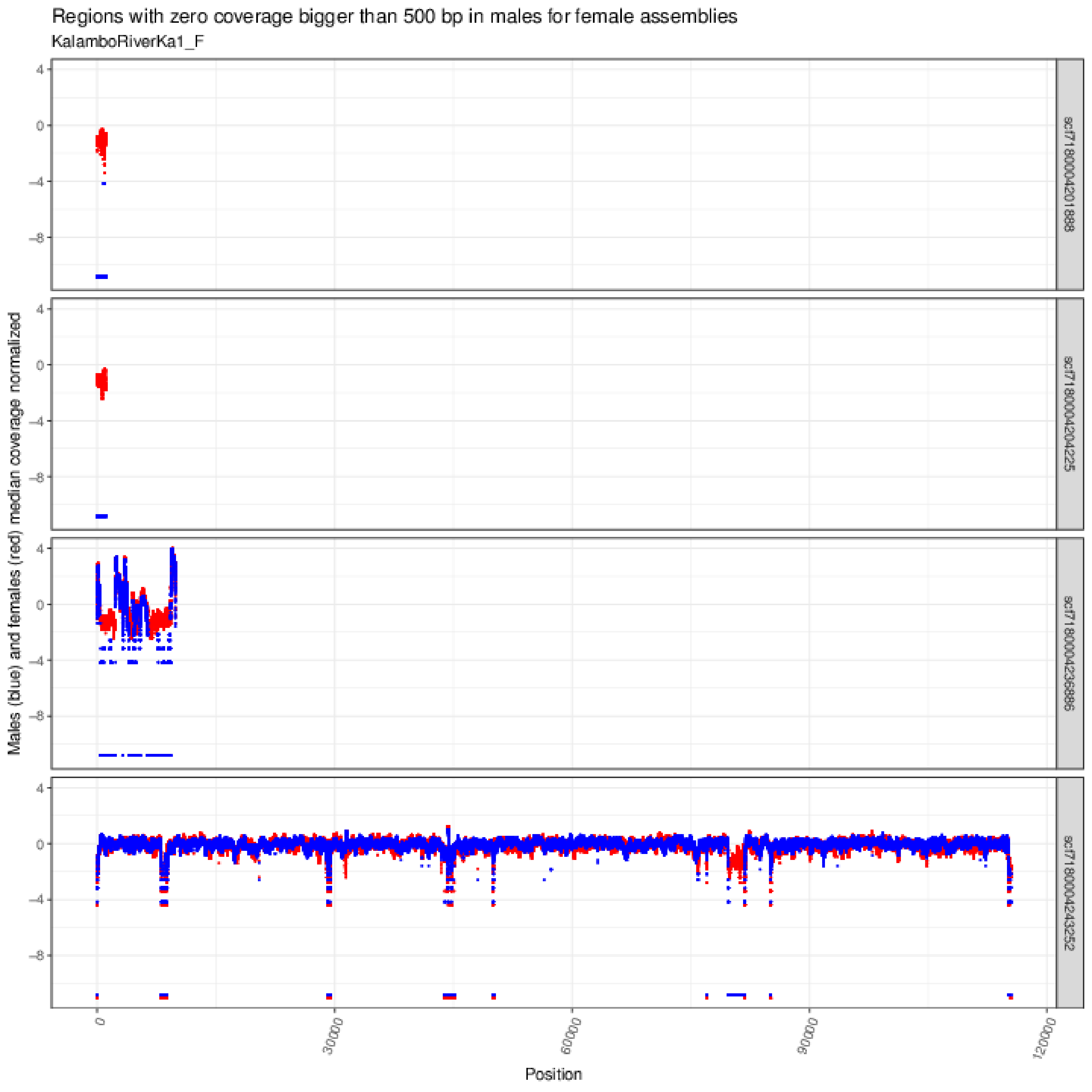
Regions with zero coverage bigger than 500 bp in males for female assemblies
KalamboRiverKa1_F
Males (blue) and females (red) median coverage normalized
Position
4
0
-4
-8
0
30000
60000
90000
120000
scf7180004201888
scf7180004204225
scf7180004236886
scf7180004243252

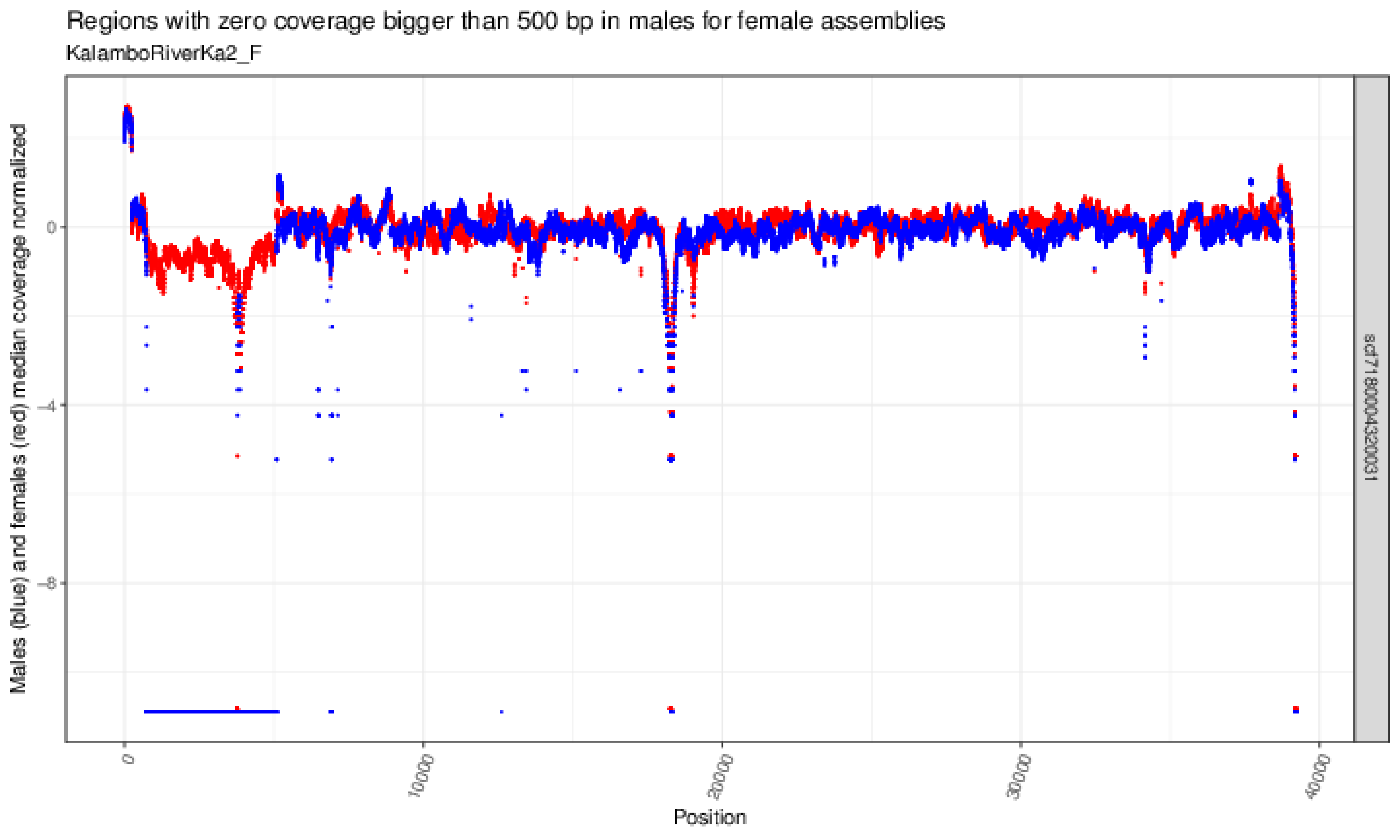
Regions with zero coverage bigger than 500 bp in males for female assemblies
KalamboRiverKa2_F
Males (blue) and females (red) median coverage normalized
0
-4
-8
0
10000
20000
30000
40000
Position
scf7180004320031

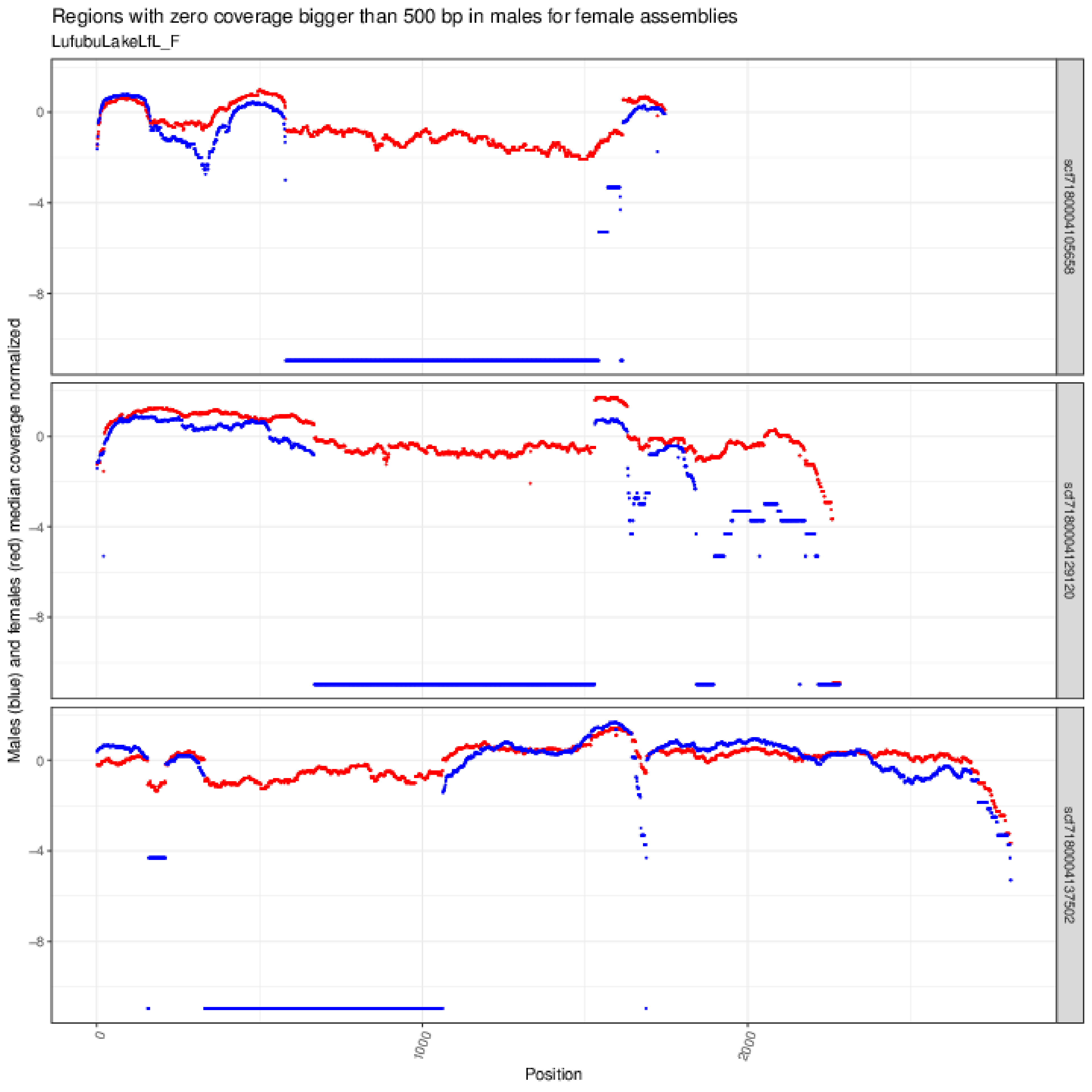

Regions with zero coverage bigger than 500 bp in males for female assemblies
LufubuLakeLfL_F
Males (blue) and females (red) median coverage normalized
Position
0
-4
-8
0
1000
2000
scf7180004105658
scf7180004129120
scf7180004137502

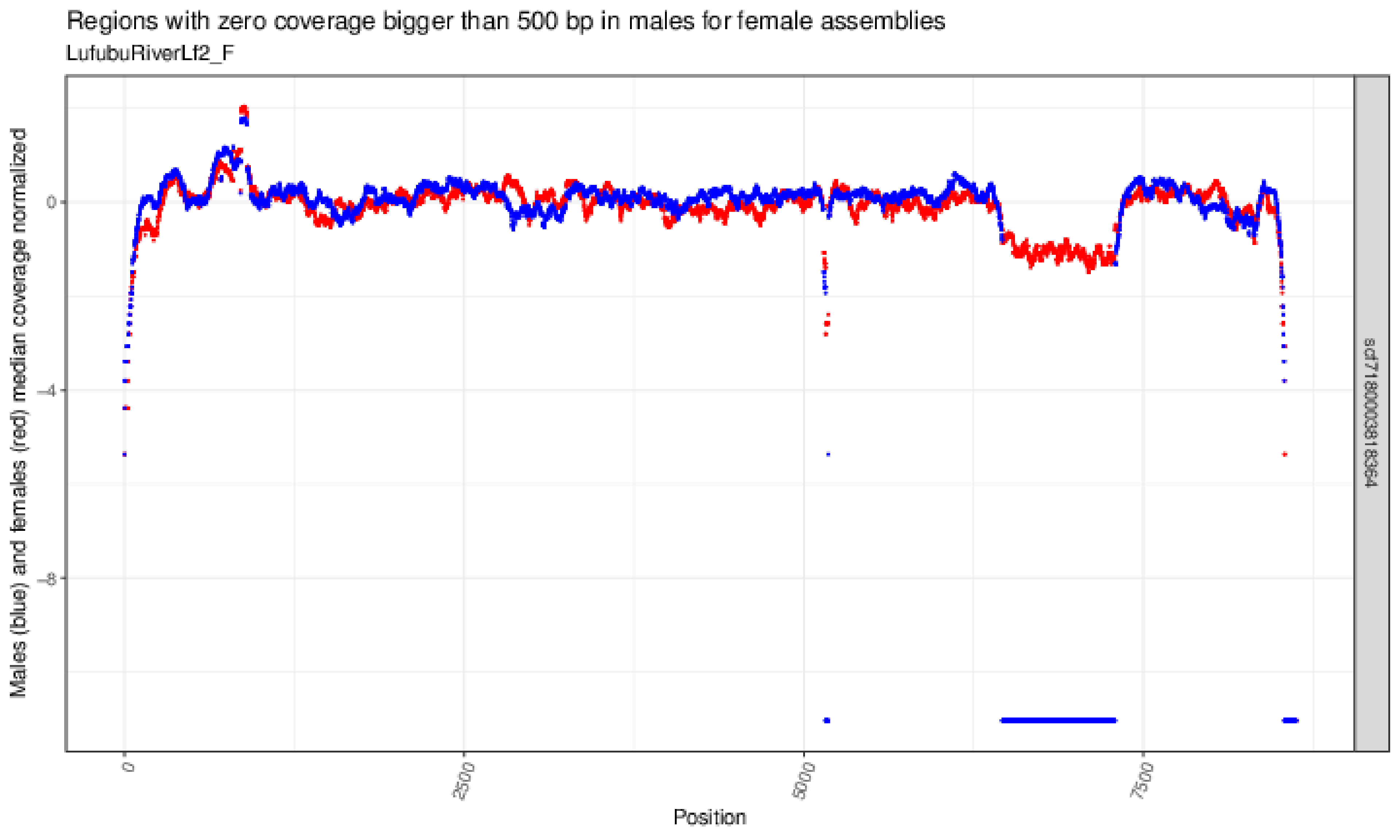
Regions with zero coverage bigger than 500 bp in males for female assemblies
LufubuRiverLf2_F
Males (blue) and females (red) median coverage normalized
0
-4
-8
0
2500
5000
7500
Position
sd71800038l8364

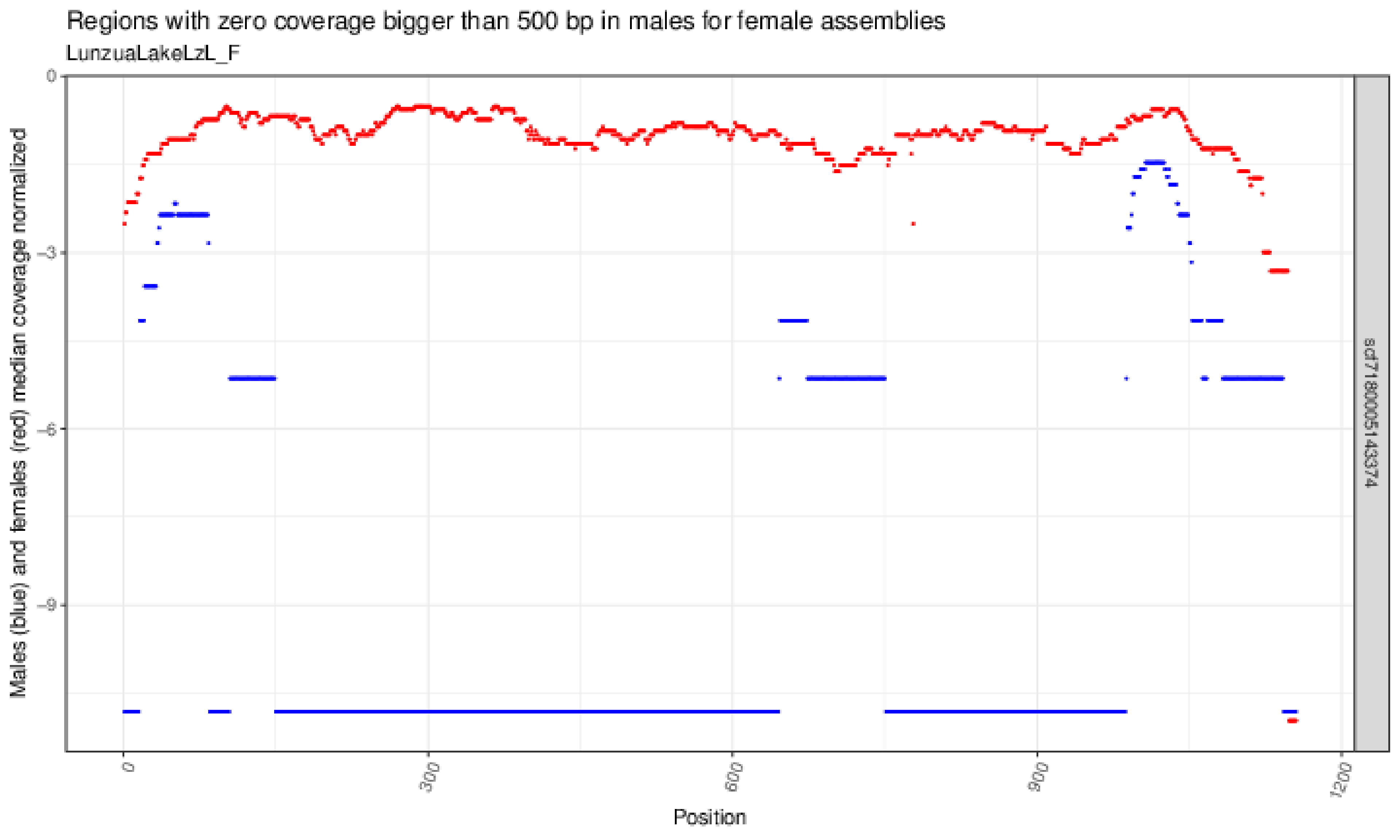
Regions with zero coverage bigger than 500 bp in males for female assemblies
LunzuaLakeLzL_F
Males (blue) and females (red) median coverage normalized
0
-3
-6
-9
0
300
600
900
1200
Position
scf7180005143374

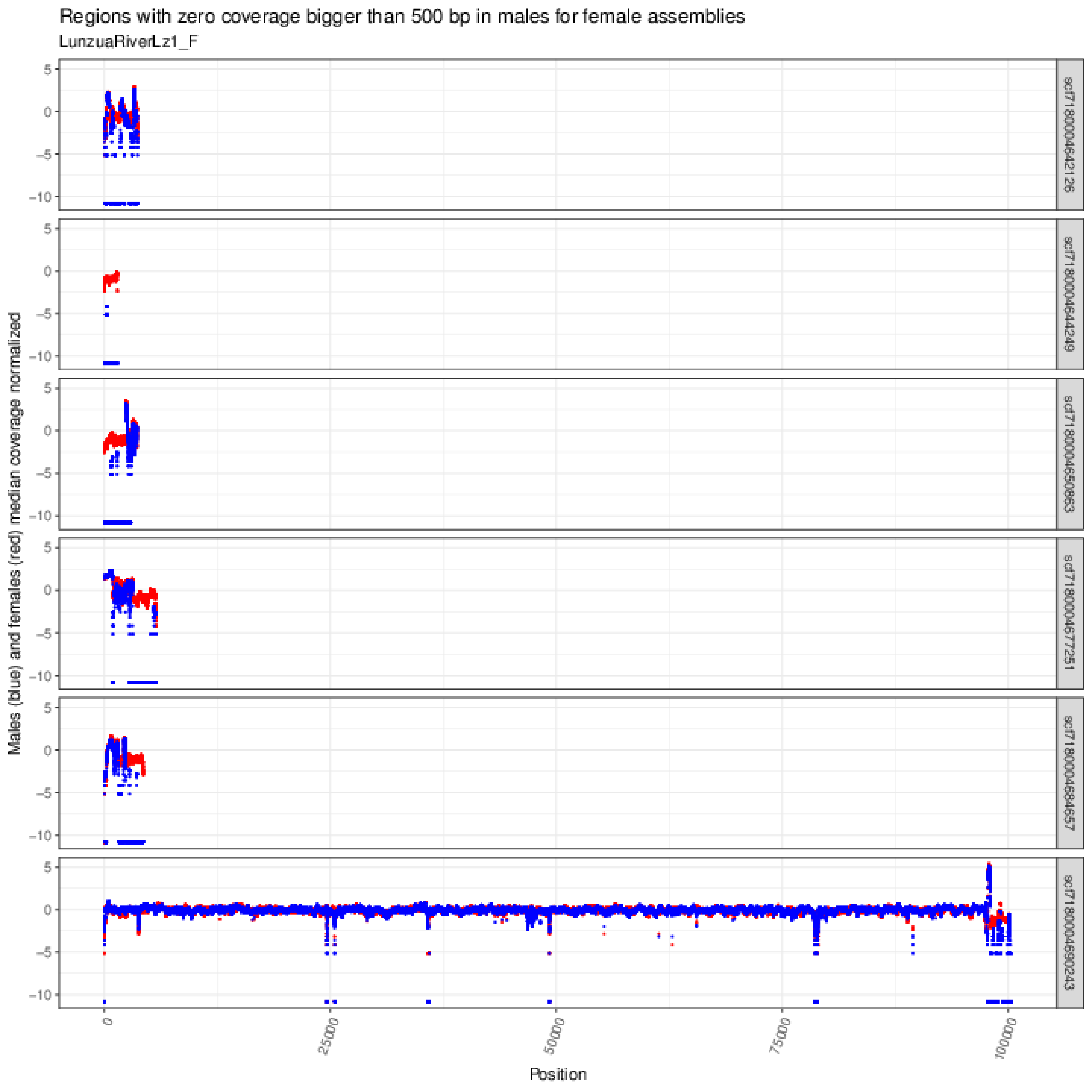

Regions with zero coverage bigger than 500 bp in males for female assemblies
LunzuaRiverLz1_F
Males (blue) and females (red) median coverage normalized
scf7180004642126
scf7180004644249
scf7180004650863
scf7180004677251
scf7180004684657
scf7180004690243
5
0
−5
−10
0
25000
50000
75000
100000
Position

Regions with zero coverage bigger than 500 bp in males for female assemblies

RuziziStreamRu1_F

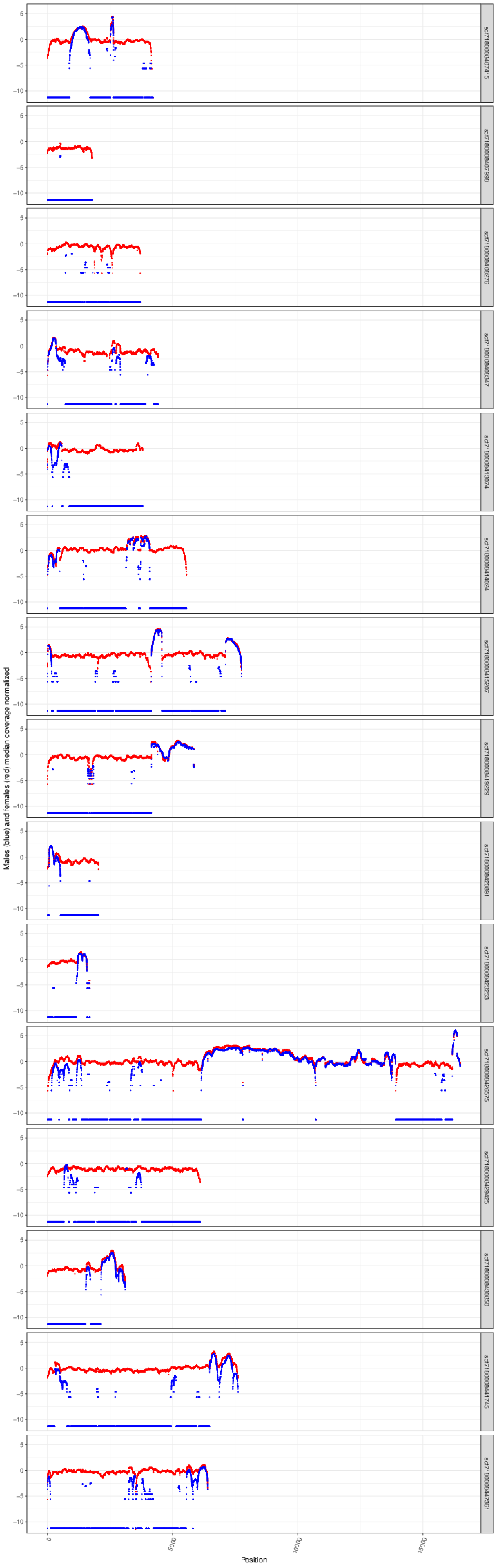

Supplement: jkad011_Supplementary_Data [file jkad011_supplementary_data.zip › File_S4.zip!/File_S3/Female-specific_regions_in_Scaffolds.pdf]
