# Supplementary material for: No evidence for sex chromosomes in natural populations of the cichlid fish Astatotilapia burtoni
Source: G3 (Bethesda). 2023 Jan 17;13(3):jkad011. doi: 10.1093/g3journal/jkad011 (PMC9997565; doi:10.1093/g3journal/jkad011)

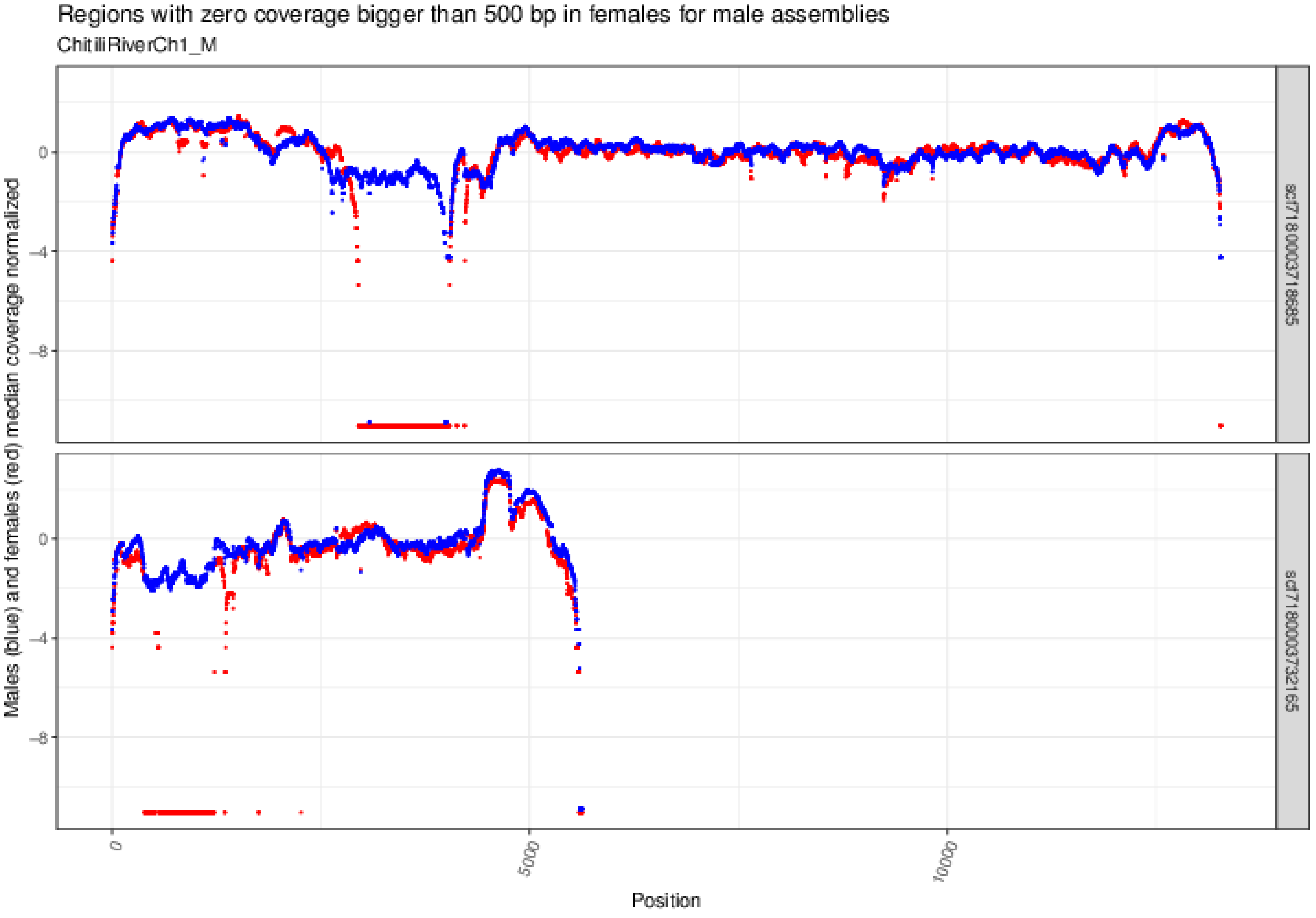
Regions with zero coverage bigger than 500 bp in females for male assemblies
ChitiliRiverCh1_M
Males (blue) and females (red) median coverage normalized
Position
0
-4
-8
0
5000
10000
scf7180003718685
scf7180003732165

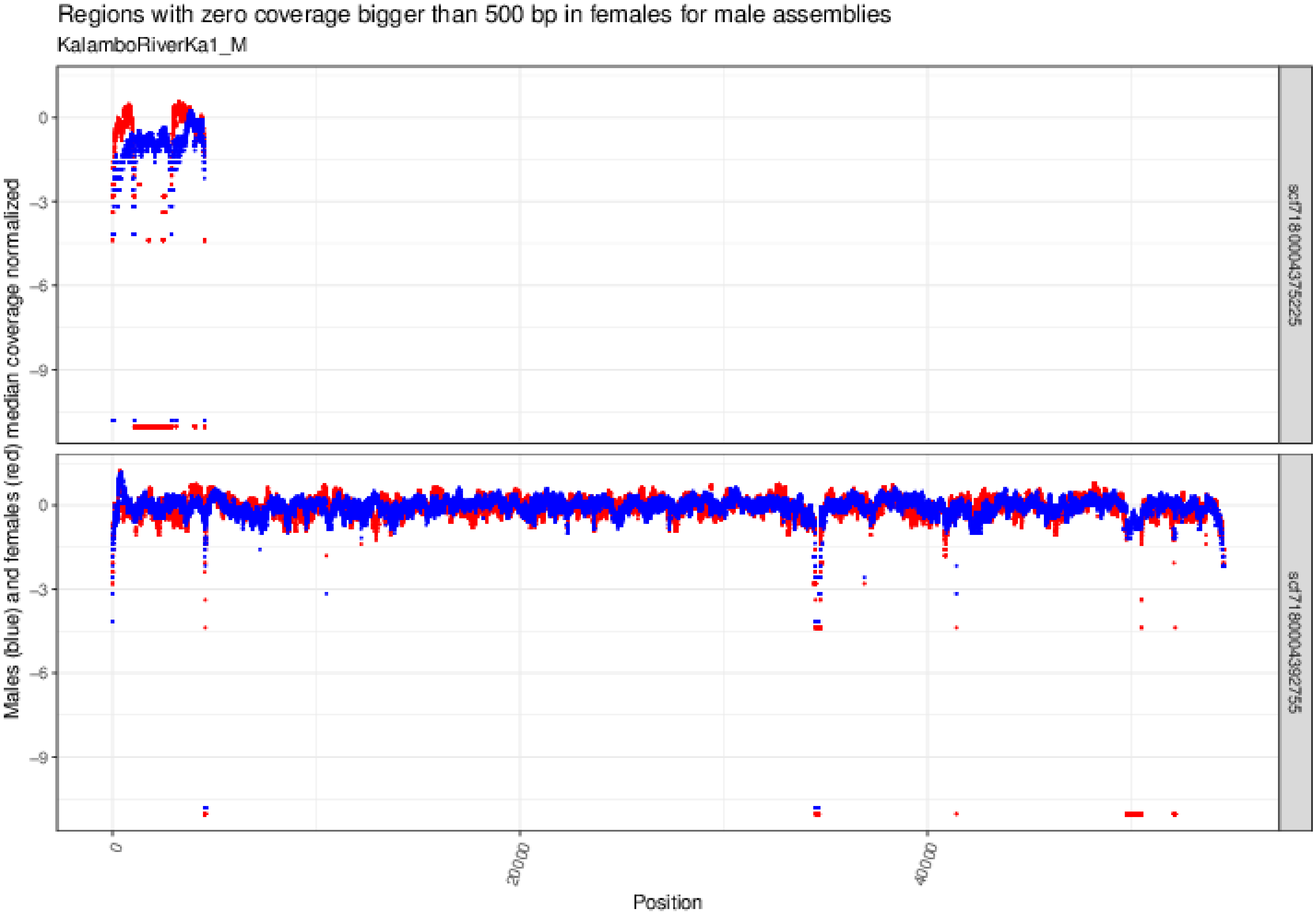
Regions with zero coverage bigger than 500 bp in females for male assemblies
KalamboRiverKa1_M
Males (blue) and females (red) median coverage normalized
0
-3
-6
-9
scf7180004375225
scf7180004392755
0
20000
40000
Position

Regions with zero coverage bigger than 500 bp in females for male assemblies

KalamboRiverKa2_M

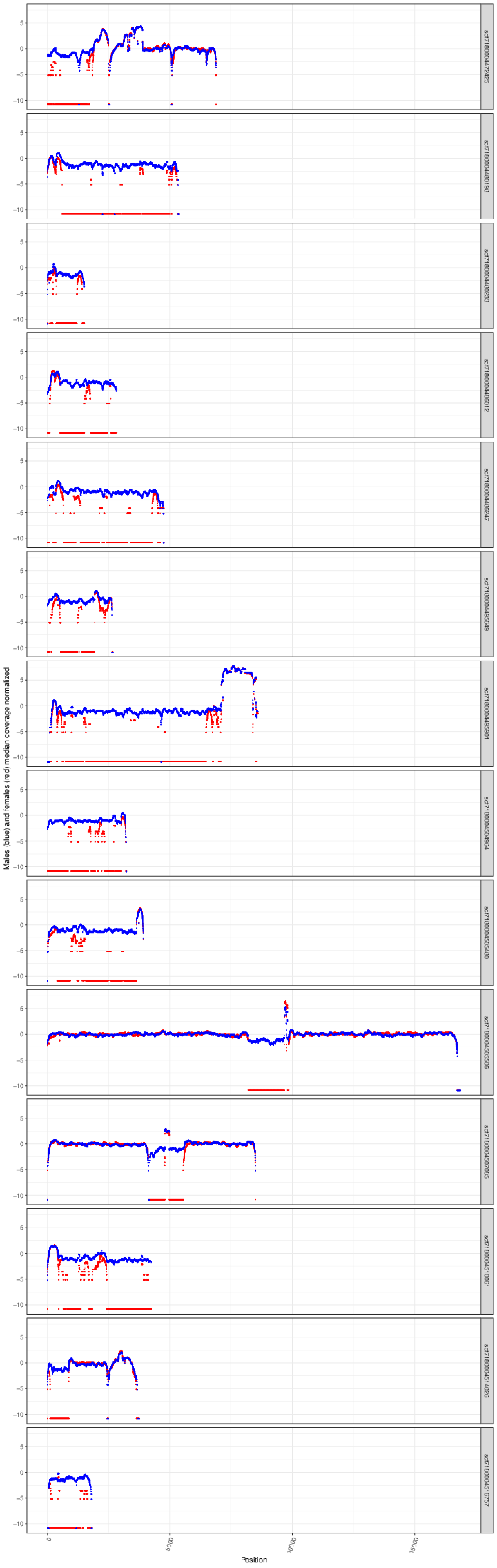

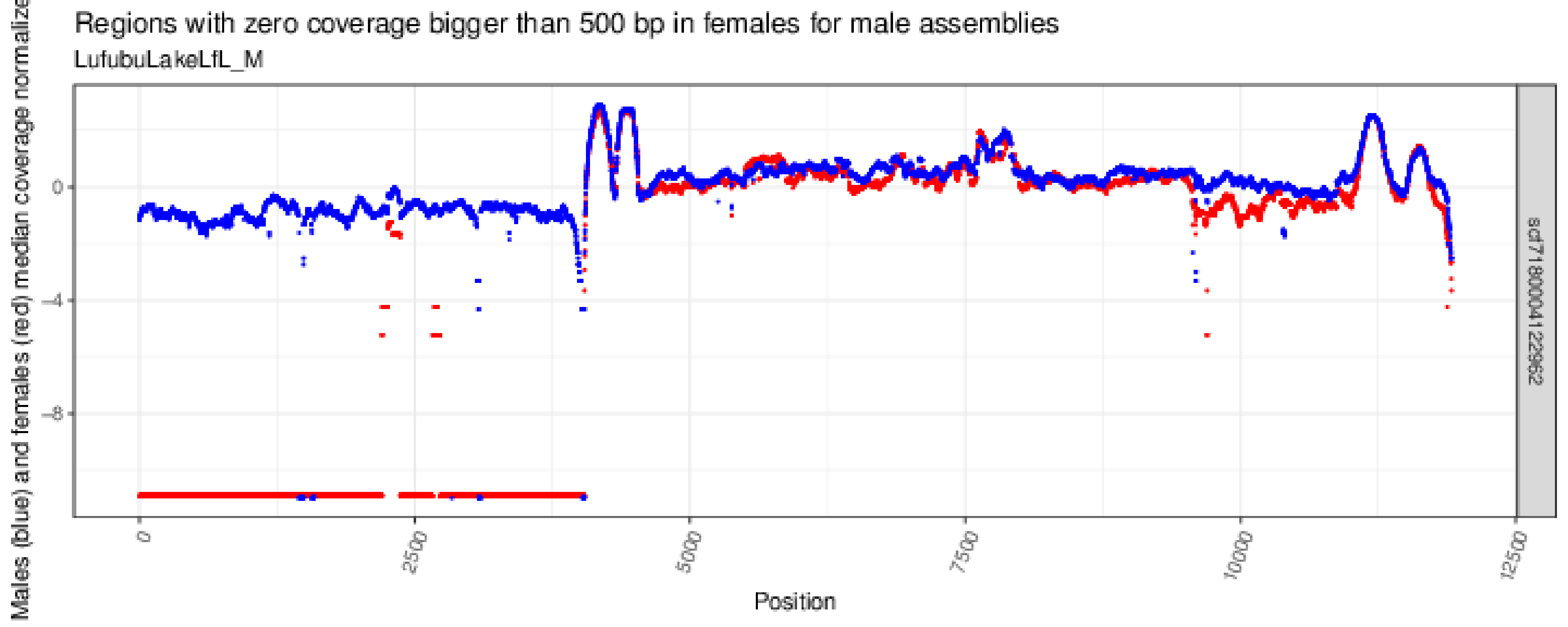

Regions with zero coverage bigger than 500 bp in females for male assemblies
LufubuLakeLfL_M
Males (blue) and females (red) median coverage normalize
0
-4
-8
0
2500
5000
7500
10000
12500
Position
scf7180004122962

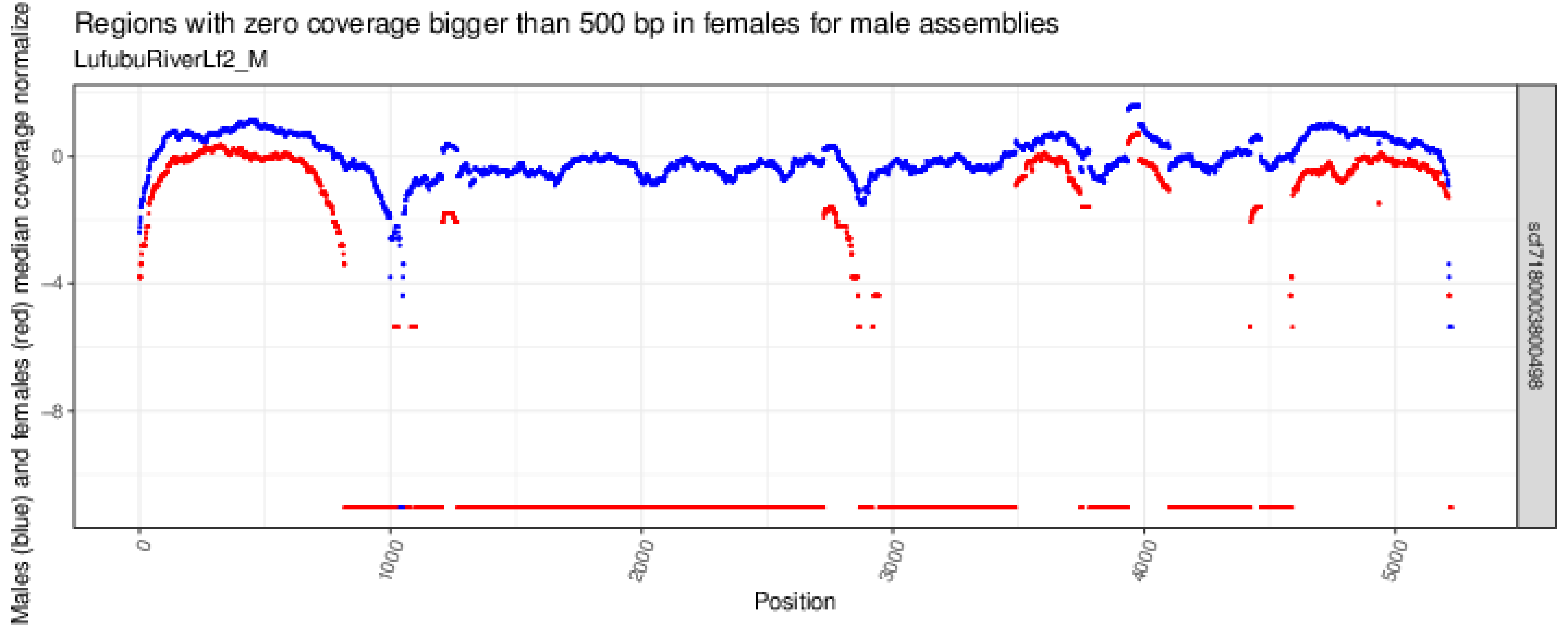

Regions with zero coverage bigger than 500 bp in females for male assemblies
LufubuRiverLf2_M
Males (blue) and females (red) median coverage normalize
0
-4
-8
0
1000
2000
3000
4000
5000
Position
scf7180003800498

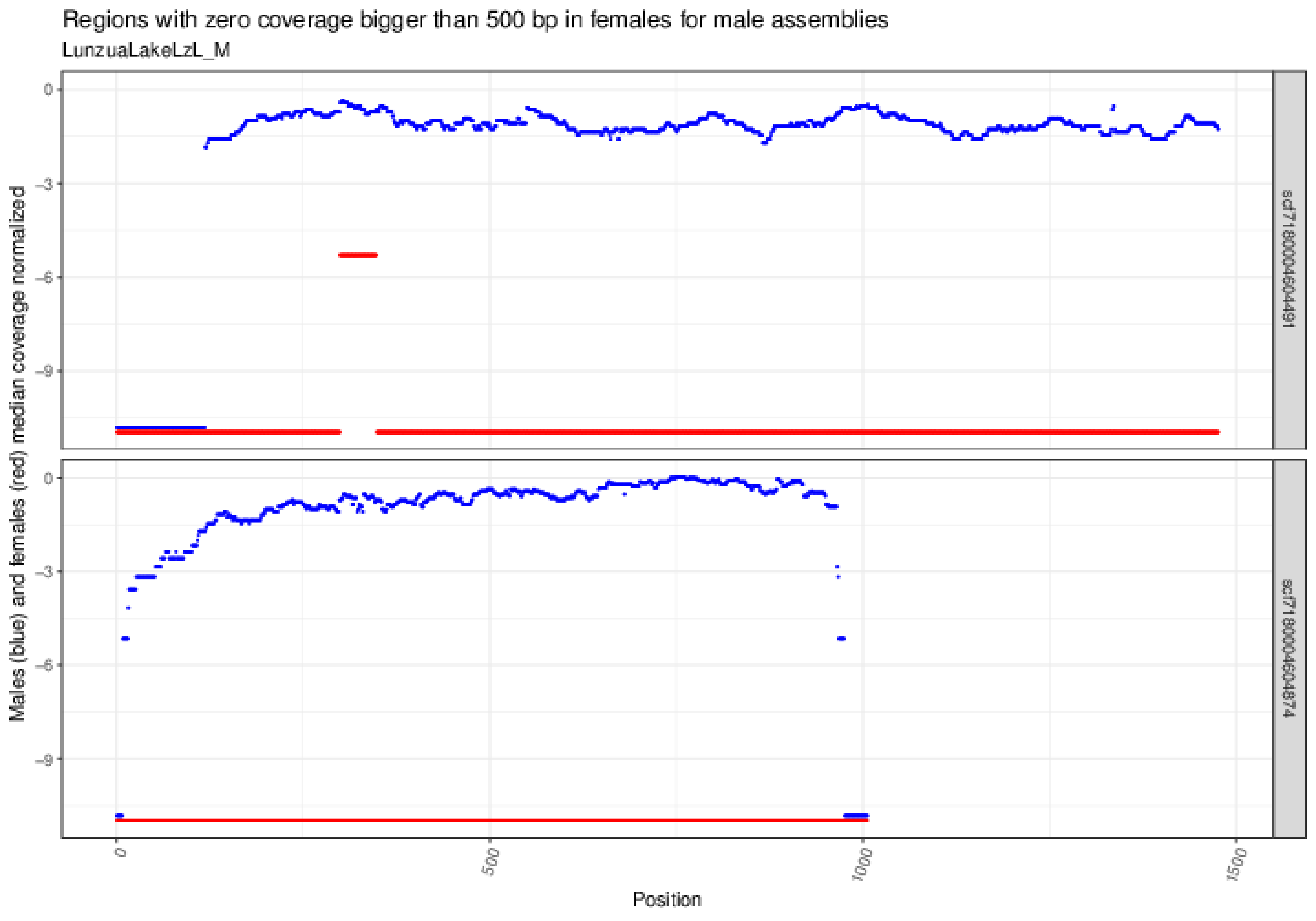

Regions with zero coverage bigger than 500 bp in females for male assemblies
LunzuaLakeLzL_M
Males (blue) and females (red) median coverage normalized
Position
0
-3
-6
-9
0
500
1000
1500
scf7180004604491
scf7180004604874

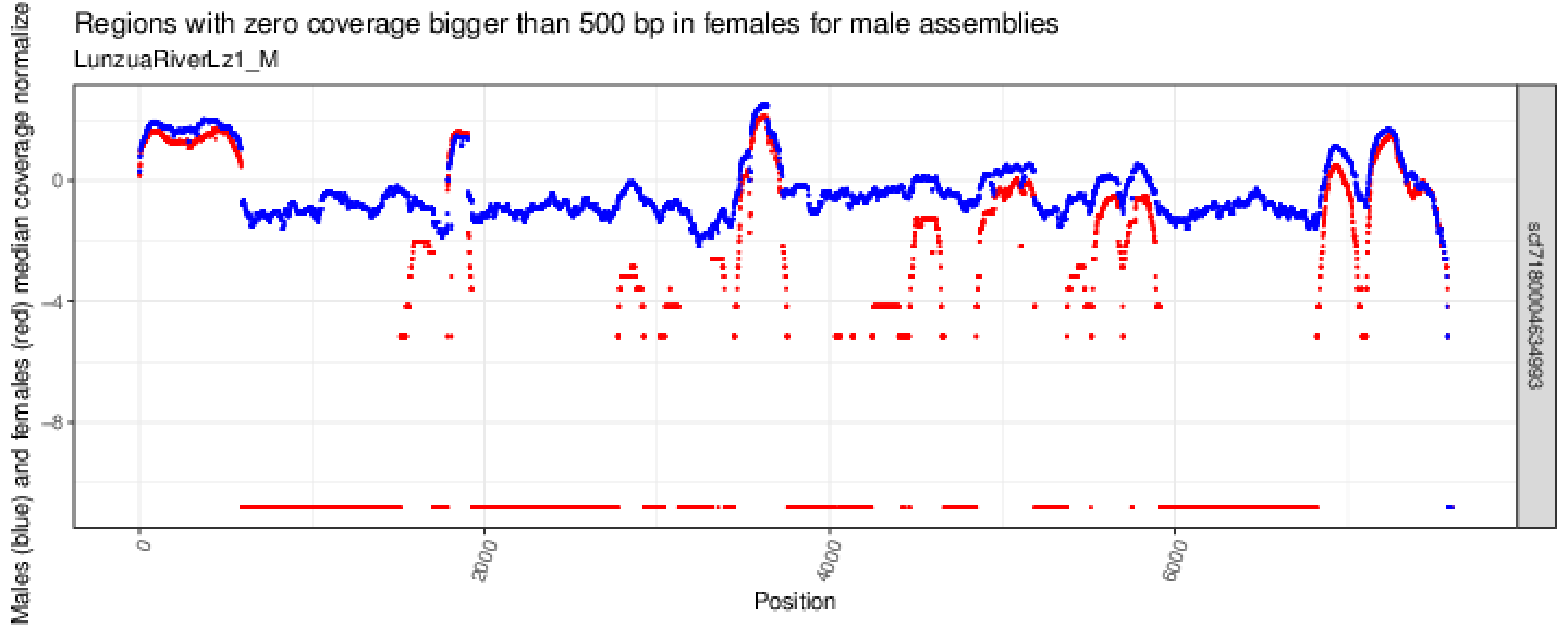

Regions with zero coverage bigger than 500 bp in females for male assemblies
LunzuaRiverLz1_M
Males (blue) and females (red) median coverage normalize
0
-4
-8
0
2000
4000
6000
Position
sd7180004634993

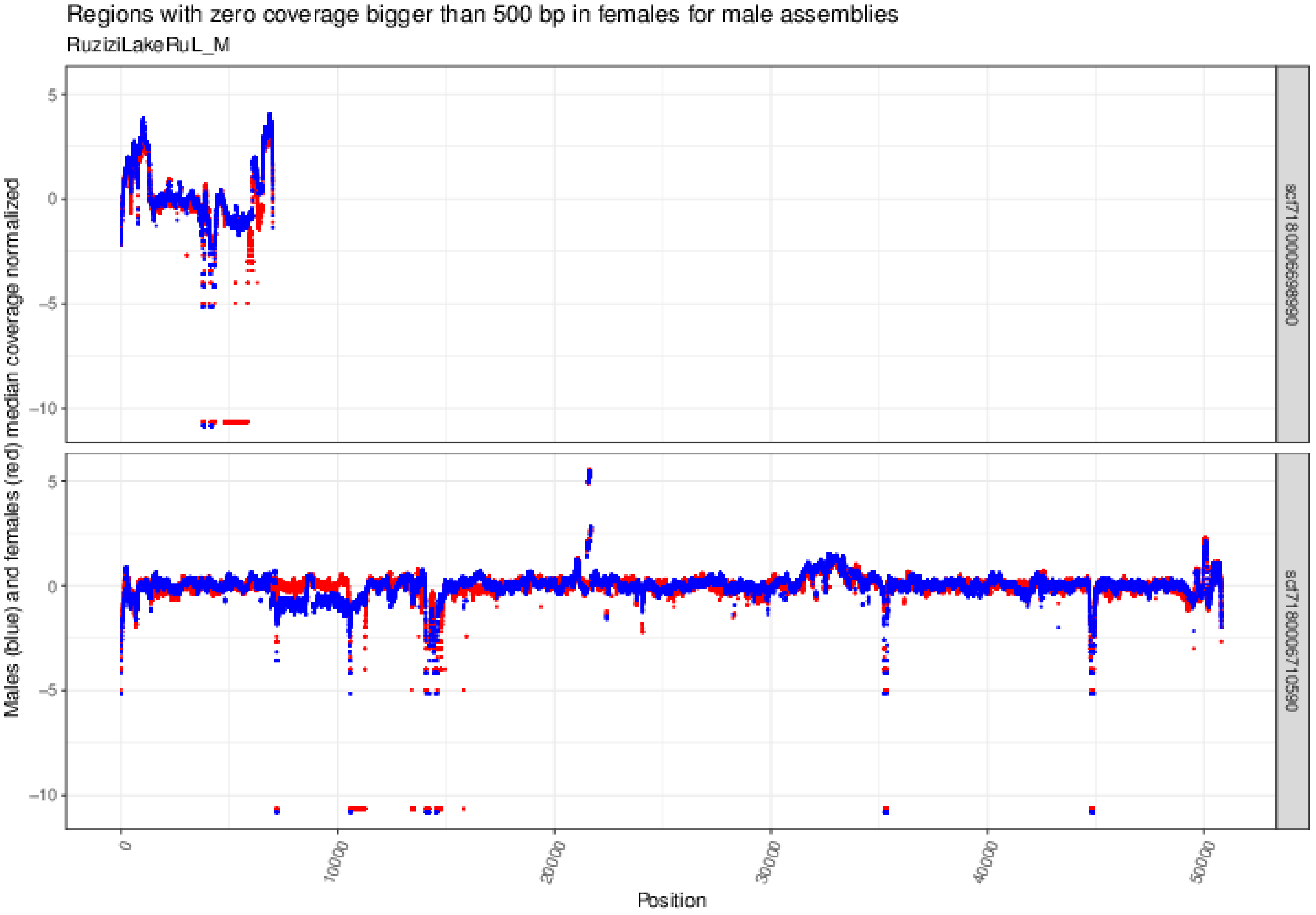
Regions with zero coverage bigger than 500 bp in females for male assemblies
RuziziLakeRuL_M
Males (blue) and females (red) median coverage normalized
5
0
−5
−10
0
10000
20000
30000
40000
50000
Position

Regions with zero coverage bigger than 500 bp in females for male assemblies

RuziziStreamRu1_M

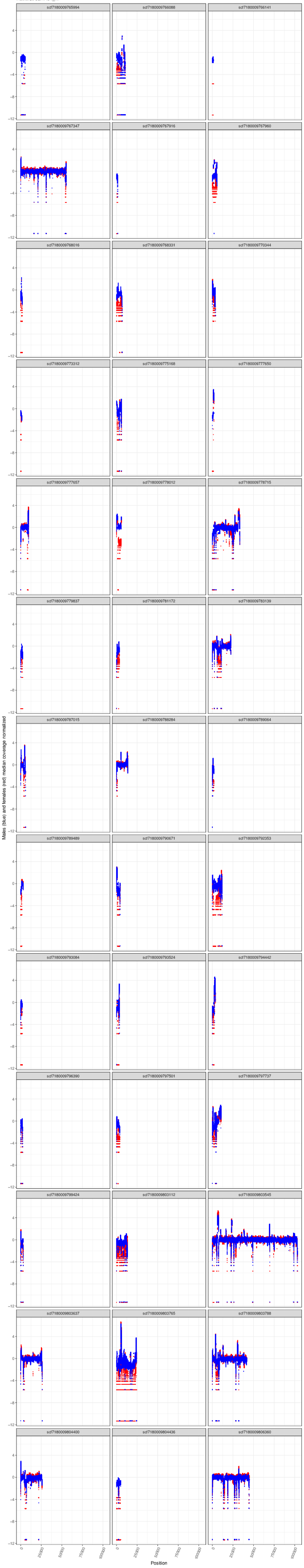

Supplement: jkad011_Supplementary_Data [file jkad011_supplementary_data.zip › File_S4.zip!/File_S3/Male-specific_regions_in_Scaffolds.pdf]
