# Supplementary material for: No evidence for sex chromosomes in natural populations of the cichlid fish Astatotilapia burtoni
Source: G3 (Bethesda). 2023 Jan 17;13(3):jkad011. doi: 10.1093/g3journal/jkad011 (PMC9997565; doi:10.1093/g3journal/jkad011)
Supplement: jkad011_Supplementary_Data [file jkad011_supplementary_data.zip › File_S4.zip!/File_S3/File_S3_README.rtf]

File S3
Female-specific_regions_in_Scaffolds.pdf
Scaffolds with female-specific regions of 500bp or more of coverage. Each separate sheet shows the scaffolds found in each population. The right-hand side labels show the assigned scaffold name. Red = female median coverage normalized by overall median depth for the corresponding population across the de novo assembled genome, blue = male median coverage normalized by overall median depth for the corresponding population across the de novo assembled genome. 
Male-specific_regions_in_Scaffolds.pdf
Scaffolds with male-specific regions of 500bp or more of coverage. Each separate sheet shows the scaffolds found in each population. The right-hand side labels show the assigned scaffold name. Red = female median coverage normalized by overall median depth for the corresponding population across the de novo assembled genome, blue = male median coverage normalized by overall median depth for the corresponding population across the de novo assembled genome.
